# Supplementary material for: Coherence distillation machines are impossible in quantum thermodynamics
Source: Nat Commun. 2020 Jan 7;11:25. doi: 10.1038/s41467-019-13846-3 (PMC6946712; doi:10.1038/s41467-019-13846-3)
Supplement: Supplementary file 1 — Supplementary Information [file 41467_2019_13846_MOESM1_ESM.pdf]

Supplementary Information:

Coherence distillation machines are impossible in quantum thermodynamics

Iman Marvian

Contents

Supplementary Note 1: Three equivalent definitions of TI operations 3
Covariant Stinespring Dilation theorem (Equivalence of statements (1) and (2) in theorem 1) 3
Completely Incoherence-Preserving operations (Equivalence of statements (1) and (3) in theorem 1) 5
General symmetries: Completely symmetry-preserving operations 6

Supplementary Note 2: Purity of Coherence 8
Properties of purity of coherence 8
Connection with Petz-Rényi relative entropy 9
Stochastic state conversions under TI operations (Proof of Eq.5 in the paper) 10
States with infinite purity of coherence 11
Purity of coherence is lower-bounded by Quantum Fisher Information 11
Purity of coherence for Qubits 12
Purity of coherence for states close to the totally mixed state 13

Supplementary Note 3: Purity of coherence for a mixed state close to a pure state 15

Supplementary Note 4: QFI and purity of coherence in the iid regime (Proof of Eq. 10 and Eq. 11 in the paper) 18
Proof of lemma 3 (A lower bound on the energy variance of pure states which are close to an iid pure state) 19
Proof of lemma 4 20

Supplementary Note 5: Extension of the main theorem: Finite helper systems do not help 23
Proof of lemma 5 23

Supplementary Note 6: Mixed states with distillable coherence 26
Generalization of the above example 28

Supplementary Note 7: Sub-linear distillation with a measure-and-prepare TI process (Proof of Eq. 14 in the paper) 29
Covariant estimators 29
A TI measure-and-prepare channel 30
Proof of Supplementary Eq.254 31

Supplementary Note 8: Purity of coherence of the output of Measure-and-Prepare TI channels is upper bounded by QFI of the input 33

Supplementary Note 9: Distillation in the single-shot regime 37
Maximum achievable fidelity with a pure state (Proof of Eq.16 in the paper) 37

Supplementary Note 10: Qubit example (Proof of Eq. 17 in the paper) 41

Supplementary Note 1: Three equivalent definitions of TI operations

In this section we review some useful properties of Time-translationally Invariant (TI) operations (See e.g. [1] for further discussion). We can summarize these properties in the following theorem.

Theorem 1 Let $\mathcal{E}$ be a Completely Positive Trace-Preserving (CPTP) linear map with arbitrary input and output spaces with Hamiltonians H_{in} and H_{out}, respectively. The following three properties are equivalent:

1. **Invariance under time-translations**: The map $\mathcal{E}$ satisfies

$$\forall t \in \mathbb{R}: \quad \mathcal{E}(e^{-iH_{\text{in}}t}\rho_{\text{in}}e^{iH_{\text{in}}t}) = e^{-iH_{\text{out}}t}\mathcal{E}(\rho_{\text{in}})e^{iH_{\text{out}}t} \ , \tag{1}$$

   for arbitrary input state ρ_{in}.

2. **Covariant Stinespring Dilation**: The map $\mathcal{E}$ can be implemented by coupling the input system to an auxiliary system A with Hamiltonian H_{A}, whose initial state $|\eta\rangle$ is an eigenstate of H_{A}, via an energy-conserving unitary U, such that

$$\mathcal{E}(\rho_{\text{in}}) = \text{Tr}_{\text{A}'}\left(U[\rho_{\text{in}} \otimes |\eta\rangle\langle\eta|_{\text{A}}]U^\dagger\right) \ , \tag{2}$$

   where (i) A' denotes the discarded output auxiliary system which is a closed system with Hamiltonian $H_{\text{A}'}$, (ii) the unitary U is energy-conserving, i.e.

$$U(H_{\text{in}} \otimes I_{\text{A}} + I_{\text{in}} \otimes H_{\text{A}}) = (H_{\text{out}} \otimes I_{\text{A}'} + I_{\text{out}} \otimes H_{\text{A}'})U \ , \tag{3}$$

   where I_{in}, I_{out}, I_{A} and $I_{\text{A}'}$ are, the identity operators on the input and output systems, and input and output auxiliary systems, respectively, and (iii) $|\eta\rangle$ is an eigenstate of H_{A}.

3. **Completely incoherence-preserving**: $\mathcal{E}$ is completely incoherence-preserving, that is for any auxiliary system B with an arbitrary Hamiltonian H_{B}, and any joint state $\rho_{\text{in,B}}$ of the input system (denoted by in) and the auxiliary system B, if $\rho_{\text{in,B}}$ is incoherent with respect to the total Hamiltonian $H_{\text{in}} \otimes I_{\text{B}} + I_{\text{in}} \otimes H_{\text{B}}$, then the corresponding output state $\mathcal{E} \otimes \mathcal{I}_{\text{B}}(\rho_{\text{in, B}})$ is also incoherent with respect to the Hamiltonian $H_{\text{out}} \otimes I_{\text{B}} + I_{\text{out}} \otimes H_{\text{B}}$, i.e.

$$[\rho_{\text{in, B}} \ , H_{\text{in}} \otimes I_{\text{B}} + I_{\text{in}} \otimes H_{\text{B}}] = 0 \implies [\mathcal{E} \otimes \mathcal{I}_{\text{B}}(\rho_{\text{in, B}}) \ , H_{\text{out}} \otimes I_{\text{B}} + I_{\text{out}} \otimes H_{\text{B}}] = 0 \ . \tag{4}$$

Remark. As we explain later, to check whether a quantum operation is completely-incoherence preserving or not, one only needs to consider this condition for a system B whose dimension is equal to d_{in}, the dimension of the input of $\mathcal{E}$. Also, one only needs to check this condition for one initial state, namely the maximally entangled state $|\Psi\rangle_{\text{in,B}} = \frac{1}{\sqrt{d_{\text{in}}}}\sum_{i=1}^{d_{\text{in}}}|ii\rangle$, with the Hamiltonian of B equal to $H_{\text{B}} = -H_{\text{A}}^T$, where the transpose is defined with respect to the basis $\{|i\rangle\}$.

The notion of completely incoherence-preserving operations can be compared with the notion of *incoherence-preserving* operations, also known as *maximally incoherent* operations. These are operations which map incoherent state of the input system S to incoherent states of the output system [2]. It turns out this set is strictly larger than the set of completely incoherence-preserving operations, i.e. there are operations which are incoherence-preserving, but not completely incoherence-preserving. For instance, any unitary transformation which permutes energy eigenstates with different energies, is an incoherence-preserving but not completely incoherence-preserving operation (The relation between the two sets in analogous to the relation between positive operations and completely positive operations).

Covariant Stinespring Dilation theorem (Equivalence of statements (1) and (2) in theorem 1)

Equivalence of properties (1) and (2), i.e. Invariance Under Time-Translation and Covariant Stinespring Dilation is proven before, e.g. in [1]. In fact, theorem 25 of [1] establishes this equivalence for a general symmetry group. For completeness, we present the proof in the case of time-translation symmetry.

First, it is straightforward to see that the existence of Covariant Stinespring Dilation implies Time-translation symmetry (Intuitively, covariant Stinespring dilation provides a method for implementing the quantum operation. Since each step in this method respects the time-translation symmetry, the composition should also respect the symmetry).

To prove statement (1) implies statement (2), we use a result of [3], which shows the Kraus representation of TI operations can be written in a special canonical form. According to this lemma, by exploiting the unitary freedom in defining the Kraus

operators of a general quantum operation [4], we can write any TI operation $\mathcal{E}_{\text{TI}}$, in the form

$$\mathcal{E}_{\text{TI}}(\sigma) = \sum_{E,\alpha} K_{(E,\alpha)} \sigma K^{\dagger}_{(E,\alpha)} \ , \tag{5}$$

where, in addition to the normalization condition $\sum_{E,\alpha} K^{\dagger}_{(E,\alpha)} K_{(E,\alpha)} = I_{\text{out}}$, Kraus operators satisfy the condition

$$e^{-iH_{\text{out}}t} K_{(E,\alpha)} e^{iH_{\text{in}}t} = e^{-iEt} K_{(E,\alpha)} \ . \tag{6}$$

For completeness, we present the proof of this result, originally proven in [3]: Consider an arbitrary Kraus decomposition $\mathcal{E}_{\text{TI}}(\cdot) = \sum_{\mu} \tilde{K}_{\mu}(\cdot)\tilde{K}^{\dagger}_{\mu}$, with linearly independent Kraus operators $\{\tilde{K}_{\mu}\}_{\mu}$. Then, invariance under time translation implies for arbitrary $t \in \mathbb{R}$, the set $\{e^{-iH_{\text{out}}t}\tilde{K}_{\mu}e^{iH_{\text{in}}t}\}_{\mu}$ also defines a valid Kraus representation of $\mathcal{E}_{\text{TI}}$. But, two sets of linearly independent Kraus operators describe the same quantum operation, if and only if they are related via a unitary, i.e. there exists a unitary transformation $V(t)$ such that, $e^{-iH_{\text{out}}t}\tilde{K}_{\mu}e^{iH_{\text{in}}t} = \sum_{\mu'} V_{\mu\mu'}(t)\tilde{K}_{\mu'}$.

This equation together with the fact that $\{\tilde{K}_{\mu}\}$ are linearly independent implies that $V(t_1)V(t_2) = V(t_1 + t_2)$ (indeed, it implies that the unitary $V(t)$ is itself a representation of the time-translation symmetry).

We conclude that there exists a unitary S which simultaneously diagonalizes all unitaries $V(t)$ for all $t \in \mathbb{R}$ and decomposes this representation to irreducible 1-dimensional representation, as $SV(t)S^{\dagger} = \sum_{(E,\alpha)} e^{-iEt}|E,\alpha\rangle\langle E,\alpha|$, where α is a multiplicity index. Define the new Kraus operators

$$K_{(E,\alpha)} \equiv \sum_{\mu} S_{(E,\alpha),\mu} \tilde{K}_{\mu} \ , \tag{7}$$

which implies

$$\tilde{K}_{\mu} = \sum_{(E,\alpha)} S^{*}_{(E,\alpha),\mu} K_{(E,\alpha)} \ , \tag{8}$$

The unitarity of S guarantees that $\sum_{E,\alpha} K^{\dagger}_{(E,\alpha)} K_{(E,\alpha)} = \sum_{\mu} K^{\dagger}_{\mu} K_{\mu} = I_{\text{out}}$. Furthermore,

$$\begin{aligned}
e^{-iH_{\text{out}}t} K_{(E,\alpha)} e^{iH_{\text{in}}t} &= \sum_{\mu} S_{(E,\alpha),\mu}\ e^{-iH_{\text{out}}t} \tilde{K}_{\mu} e^{iH_{\text{in}}t} && (9)\\
&= \sum_{\mu} S_{(E,\alpha),\mu} \sum_{\mu'} V_{\mu\mu'}(t) \tilde{K}_{\mu'} && (10)\\
&= \sum_{\mu} S_{(E,\alpha),\mu} \sum_{\mu'} V_{\mu\mu'}(t) \sum_{(E',\alpha')} S^{*}_{(E',\alpha'),\mu'} K_{(E',\alpha')} && (11)\\
&= \sum_{(E',\alpha')} K_{(E',\alpha')} \sum_{\mu,\mu'} S_{(E,\alpha),\mu} V_{\mu\mu'}(t) S^{*}_{(E',\alpha'),\mu'} && (12)\\
&= \sum_{(E',\alpha')} K_{(E',\alpha')} e^{-iEt}\ \delta_{E,E'} \delta_{\alpha,\alpha'} && (13)\\
&= K_{(E,\alpha)} e^{-iEt} \ , && (14)
\end{aligned}$$

where, we have used Supplementary Eq.(7) to get the first line, and Supplementary Eq.(8) to get the third line.

Next, we use this result to construct an energy-conserving unitary which implements $\mathcal{E}_{\text{TI}}$. Define the operator

$$W = \sum_{(E,\alpha)} K_{(E,\alpha)} \otimes |E,\alpha\rangle\langle\eta| \ , \tag{15}$$

where $\{|E,\alpha\rangle\}$ is an arbitrary set of orthogonal states of system A′, and $|\eta\rangle$ is an arbitrary state of A. Then, one can easily see that:

- Using the fact that $\sum_{E,\alpha} K^{\dagger}_{(E,\alpha)} K_{(E,\alpha)} = I_{\text{in}}$, we find W is an isometry, i.e.

$$W^{\dagger} W = I_{\text{in}} \otimes |\eta\rangle\langle\eta|_{\text{A}} \ , \tag{16}$$

- Isometry W is an Stinespring dilation of $\mathcal{E}_{\text{TI}}$, i.e.

$$\mathcal{E}_{\text{TI}}(\rho) = \text{Tr}_{\text{A}'}(W[\rho \otimes |\eta\rangle\langle\eta|_{\text{A}}]W^\dagger) \ . \tag{17}$$

- Suppose we define H_{A}, the Hamiltonian of A, to be a Hermitian operator with state $|\eta\rangle$ as its eigenvector with eigenvalue zero, such that $H_{\text{A}}|\eta\rangle = 0$, and $H_{\text{A}'}$ as a Hermitian operator with eigenvectors $|E,\alpha\rangle$, such that $H_{\text{A}'}|E,\alpha\rangle = -E|E,\alpha\rangle$, then

$$(e^{-iH_{\text{out}}t} \otimes e^{-iH_{\text{A}'}t})W(e^{iH_{\text{in}}t} \otimes e^{iH_{\text{A}}t}) = \sum_{(\mu,\alpha)} e^{-iH_{\text{out}}t}K_{(E,\alpha)}e^{iH_{\text{in}}t} \otimes e^{-iH_{\text{A}'}t}|E,\alpha\rangle\langle\eta|e^{iH_{\text{A}}t} \tag{18}$$

$$= \sum_{(\mu,\alpha)} e^{-iEt}K_{(E,\alpha)} \otimes |E,\alpha\rangle\langle\eta|e^{iEt} \tag{19}$$

$$= W \ , \tag{20}$$

where to get the second line we have used Supplementary Eq.(6). Equivalently, this implies

$$(e^{-iH_{\text{out}}t} \otimes e^{-iH_{\text{A}'}t})W = W(e^{iH_{\text{in}}t} \otimes e^{iH_{\text{A}}t}) \ . \tag{21}$$

Taking the derivative with respect to t this implies,

$$W(H_{\text{in}} \otimes I_{\text{A}} + I_{\text{in}} \otimes H_{\text{A}}) = (H_{\text{out}} \otimes I_{\text{A}'} + I_{\text{out}} \otimes H_{\text{A}'})W \ . \tag{22}$$

It can be easily shown that the isometry W can always be extended to a unitary which is also energy-conserving (See [1]). This completes the proof that (1) implies (2).

Completely Incoherence-Preserving operations (Equivalence of statements (1) and (3) in theorem 1)

The fact that any TI operation is completely incoherence-preserving, follows immediately from the covariance condition in Supplementary Eq.(1). In the following we prove any completely incoherence-preserving operation is TI. To prove this we consider an auxiliary system with dimension equal to the input space of $\mathcal{E}$, and with the Hamiltonian $H_{\text{B}} = -H_{\text{in}}^T$, where T denotes transpose in an orthonormal basis $\{|i\rangle\}$. Then, consider maximally entangled state

$$|\Psi\rangle_{\text{in,B}} = \frac{1}{\sqrt{d_{\text{in}}}} \sum_{i=1}^{d_{\text{in}}} |ii\rangle \ , \tag{23}$$

where d_{in} is the dimension of the input space of $\mathcal{E}$.

It can be easily seen that this state is incoherent with respect to the total Hamiltonian

$$H_{\text{tot}} = H_{\text{in}} \otimes I_{\text{B}} + I_{\text{in}} \otimes H_{\text{B}} = H_{\text{in}} \otimes I_{\text{A}} - I_{\text{in}} \otimes H_{\text{in}}^T \tag{24}$$

This can be seen, for instance, by noting that

$$e^{-iH_{\text{tot}}t}|\Psi\rangle_{\text{in,B}} = (e^{-iH_{\text{in}}t} \otimes e^{iH_{\text{in}}^T t})|\Psi\rangle_{\text{in,B}} = (e^{-iH_{\text{in}}t} \otimes e^{iH_{\text{in}}^T t})\frac{1}{\sqrt{d_{\text{in}}}}\sum_{i=1}^{d_{\text{in}}}|ii\rangle_{\text{in,B}} = (e^{iH_{\text{in}}t}e^{-iH_{\text{in}}t} \otimes I)|\Psi\rangle_{\text{in,B}} = |\Psi\rangle_{\text{in,B}} \ , \tag{25}$$

where we have used the fact that for any operator X, $(I \otimes X)\sum_{i=1}^{d_{\text{in}}}|ii\rangle = (X^T \otimes I)\sum_{i=1}^{d_{\text{in}}}|ii\rangle$.

Therefore, since state $|\Psi\rangle_{\text{in,B}}$ is incoherent and the quantum operation $\mathcal{E}$ is, by assumption, completely incoherence-preserving, then state $\mathcal{E} \otimes \mathcal{I}_{\text{B}}(|\Psi\rangle\langle\Psi|_{\text{in,B}})$ is also incoherent, i.e.

$$[H_{\text{out}} \otimes I_{\text{B}} + I_{\text{out}} \otimes H_{\text{B}} \ , \ \mathcal{E} \otimes \mathcal{I}_{\text{B}}(|\Psi\rangle\langle\Psi|_{\text{in,B}})] = 0 \ , \tag{26}$$

where $\mathcal{I}_{\text{B}}$ is the identity operation on system B. This implies

$$[e^{-iH_{\text{out}}t} \otimes e^{-iH_{\text{B}}t}]\mathcal{E} \otimes \mathcal{I}_{\text{B}}(|\Psi\rangle\langle\Psi|_{\text{in,B}})[e^{iH_{\text{out}}t} \otimes e^{iH_{\text{B}}t}] = [e^{-iH_{\text{out}}t} \otimes e^{iH_{\text{in}}^T t}]\mathcal{E} \otimes \mathcal{I}_{\text{B}}(|\Psi\rangle\langle\Psi|_{\text{in,B}})[e^{iH_{\text{out}}t} \otimes e^{-iH_{\text{in}}^T t}] \tag{27a}$$

$$= \mathcal{E} \otimes \mathcal{I}_{\text{B}}(|\Psi\rangle\langle\Psi|_{\text{in,B}}) \ , \tag{27b}$$

for all $t \in \mathbb{R}$. Recall that $\mathcal{I}_{\rm B}$ is the identity operation on system B, and therefore, the left-hand side can be written as

$$[e^{-iH_{\rm out}t}\otimes I_{\rm B}]\mathcal{E}\otimes\mathcal{I}_{\rm B}\left([I_{\rm in}\otimes e^{-iH_{\rm B}t}]|\Psi\rangle\langle\Psi|_{\rm in,B}[I_{\rm in}\otimes e^{iH_{\rm B}t}]\right)[e^{iH_{\rm out}t}\otimes I_{\rm B}]\ . \tag{28}$$

Then, using the fact that $H_{\rm B}=-H_{\rm in}^T$ and the identity $(I\otimes X)\sum_{i=1}^{d_{\rm in}}|ii\rangle=(X^T\otimes I)\sum_{i=1}^{d_{\rm in}}|ii\rangle$, we find that the left-hand side of Supplementary Eq.(27) can be rewritten as

$$[e^{-iH_{\rm out}t}\otimes I]\mathcal{E}\otimes\mathcal{I}\big([e^{iH_{\rm in}t}\otimes I]|\Psi\rangle\langle\Psi|_{\rm in,B}[e^{-iH_{\rm in}t}\otimes I]\big)[e^{iH_{\rm out}t}\otimes I]=\mathcal{E}_t\otimes\mathcal{I}_{\rm B}(|\Psi\rangle\langle\Psi|_{\rm in,B})\ , \tag{29}$$

where we have defined the quantum operation $\mathcal{E}_t$ to be the time-translated version of $\mathcal{E}$, i.e.

$$\mathcal{E}_t(\sigma)\equiv e^{-iH_{\rm out}t}\mathcal{E}\big(e^{iH_{\rm in}t}\sigma e^{-iH_{\rm in}t}\big)e^{iH_{\rm out}t}\ . \tag{30}$$

Then, Supplementary Eq.(27) can be rewritten as

$$\forall t\in\mathbb{R}:\ \ \mathcal{E}_t\otimes\mathcal{I}_{\rm B}(|\Psi\rangle\langle\Psi|_{\rm in,B})=\mathcal{E}\otimes\mathcal{I}_{\rm B}(|\Psi\rangle\langle\Psi|_{\rm in,B})\ . \tag{31}$$

In other words, the Choi matrix of quantum operations $\mathcal{E}_t$ is independent of t. It is well-known that the Choi matrix uniquely determines the quantum operation. This immediately implies that

$$\forall t\in\mathbb{R}:\ \ \mathcal{E}_t=\mathcal{E}\ , \tag{32}$$

which means $\mathcal{E}$ is a TI operation. This completes the proof.

General symmetries: Completely symmetry-preserving operations

In this paper, we study the notion of coherence as asymmetry with respect to time-translation symmetry. This is a specific type of asymmetry. The resource theory of asymmetry studies asymmetry with respect to am arbitrary symmetry group G. In this recourse theory, one studies the consequences of restriction to quantum operations which satisfy the following covariance condition

$$\forall g\in G:\ \ U_{\rm B}(g)(\mathcal{E}(\cdot)U_{\rm B}^\dagger(g)=\mathcal{E}(U_{\rm A}(g)(\cdot)U_{\rm A}^\dagger(g))\ , \tag{33}$$

where $\mathcal{E}$ be a quantum operation from the input system A to the output system B with the unitary representation $G\ni g\to U_{\rm A,B}(g)$ on the input A and output B.

Any operation $\mathcal{E}$ which satisfies this condition is called a *covariant* or *symmetric* operation. This definition is a natural generalization of the notion of TI operations.

Similarly, we can define a generalization of the notion of completely incoherence-preserving operations: Let C be an arbitrary auxiliary system with arbitrary representation of symmetry, $G\ni g\to U_{\rm C}(g)$. We say quantum operation $\mathcal{E}$ from A to B is a *completely symmetry-preserving* (with respect to group G), if for all such auxiliary systems, and for all symmetric input states $\rho_{\rm AC}$, it holds that

$$\forall g\in G:\ [\rho_{\rm AC},U_{\rm A}(g)\otimes U_{\rm C}(g)]=0,\ \implies\forall g\in G:\ [\mathcal{E}\otimes\mathcal{I}_{\rm C}(\rho_{\rm AC}),U_{\rm B}(g)\otimes U_{\rm C}(g)]=0\ , \tag{34}$$

where $\mathcal{I}_{\rm C}$ is the identity quantum operation on system C. This condition means that any symmetric input state of systems A and C is mapped to a symmetric state of systems B and C.

Proposition An operation is completely symmetry-preserving if and only if it is covariant.

Proof. Obviously any covariant operation is non-asymmetry-generating. To see the other direction, suppose the auxiliary system C has dimension equal to the dimension of the input system A, and the representation of symmetry on C is the complex conjugate of the representation of symmetry on A, i.e. $\forall g\in G:\ U_{\rm C}(g)=\overline{U}_{\rm A}(g)$. Consider the *singlet* state

$$|\Psi\rangle_{\rm AC}=\frac{1}{\sqrt{d_{\rm A}}}\sum_{i=1}^{d_{\rm A}}|ii\rangle_{\rm AC}\ . \tag{35}$$

This state is invariant under the action of symmetry, i.e.

$$[U_{\rm A}(g)\otimes U_{\rm C}(g)]|\Psi\rangle_{\rm AC}=[U_{\rm A}(g)\otimes\overline{U}_{\rm A}(g)]|\Psi\rangle_{\rm AC}=|\Psi\rangle_{\rm AC}\ . \tag{36}$$

Now for the initial state $|\Psi\rangle_{\text{AC}}$, suppose we act on system A with quantum operation $\mathcal{E}$ and obtain state

$$\sigma_{\text{BC}} = \mathcal{E} \otimes \mathcal{I}_{\text{C}}(|\Psi\rangle\langle\Psi|_{\text{AC}}) \ . \tag{37}$$

Then, the fact that $\mathcal{E}$ is non-asymmetry-generating, implies σ_{BC} does not break the symmetry, i.e.

$$\forall g \in G: \ \ [U_{\text{B}}(g) \otimes U_{\text{C}}(g)]\sigma_{\text{BC}}[U_{\text{B}}^{\dagger}(g) \otimes U_{\text{C}}^{\dagger}(g)] = \sigma_{\text{BC}} \ . \tag{38}$$

This implies

$$\begin{aligned}
\forall g \in G: \ \ \mathcal{E} \otimes \mathcal{I}_{\text{C}}(|\Psi\rangle\langle\Psi|_{\text{AC}}) &= [U_{\text{B}}(g) \otimes U_{\text{C}}(g)]\mathcal{E} \otimes \mathcal{I}_{\text{C}}(|\Psi\rangle\langle\Psi|_{\text{AC}})[U_{\text{B}}^{\dagger}(g) \otimes U_{\text{C}}^{\dagger}(g)] && (39)\\
&= [U_{\text{B}}(g) \otimes I_{\text{C}}]\mathcal{E} \otimes \mathcal{I}_{\text{C}}([I_{\text{A}} \otimes U_{\text{C}}(g)]|\Psi\rangle\langle\Psi|_{\text{AC}}[I_{\text{A}} \otimes U_{\text{C}}^{\dagger}(g)])[U_{\text{B}}^{\dagger}(g) \otimes I_{\text{C}}] && (40)\\
&= [U_{\text{B}}(g) \otimes I_{\text{C}}]\mathcal{E} \otimes \mathcal{I}_{\text{C}}([U_{\text{A}}^{\dagger}(g) \otimes I_{\text{C}}]|\Psi\rangle\langle\Psi|_{\text{AC}}[U_{\text{A}}(g) \otimes I_{\text{C}}])[U_{\text{B}}^{\dagger}(g) \otimes I_{\text{C}}] && (41)\\
&= \mathcal{E}_g \otimes \mathcal{I}_{\text{C}}(|\Psi\rangle\langle\Psi|_{\text{AC}}) && (42)
\end{aligned}$$

where we have defined the map $\mathcal{E}_g$ to be the rotated the version of $\mathcal{E}$, such that

$$\mathcal{E}_g(\rho) = U_{\text{B}}(g)\mathcal{E}(U_{\text{A}}^{\dagger}(g)\rho U_{\text{A}}(g))U_{\text{B}}^{\dagger}(g) \tag{43}$$

for all ρ. But, because $|\Psi\rangle_{\text{AC}}$ is a maximally entangled state, this implies that

$$\forall g \in G: \ \mathcal{E}_g = \mathcal{E}. \tag{44}$$

which implies $\mathcal{E}$ is a covariant map.

□

Supplementary Note 2: Purity of Coherence

In this paper we introduce a new measure of asymmetry, which we call it *Purity of Coherence*. The purity of coherence, with respect to the eigenbasis of an observable H, is defined by

$$P_H(\rho) \equiv \text{Tr}(H\rho^2 H\rho^{-1}) - \text{Tr}(\rho H^2) \tag{45}$$

if sup$(H\rho H) \subseteq$ sup(ρ), and $P_H(\rho) = \infty$ otherwise (Note that sup$(H\rho H) =$ sup$(H\rho^2 H)$).

Equivalently,

$$P_H(\rho) = f_H(\rho) - f_H(\mathcal{D}(\rho)) \ , \tag{46}$$

where for any state σ

$$f_H(\sigma) \equiv \text{Tr}(H\sigma^2 H\sigma^{-1}) \ , \tag{47}$$

and

$$\mathcal{D}(\rho) = \sum_n P_n \rho P_n = \lim_{T\to\infty} \frac{1}{T} \int_0^T dt\ e^{-iHt} \rho e^{iHt} \ , \tag{48}$$

is the map that dephases ρ in the eigen-basis of $H = \sum_n E_n P_n$ (also known as the resource-destroying map [5]).

Using the spectral decomposition of state ρ, as $\rho = \sum_j p_j |\psi_j\rangle\langle\psi_j|$, we can rewrite the formula for the purity of coherence as

$$P_H(\rho) = \sum_{j,k} \frac{p_k^2 - p_j^2}{p_j} \ |\langle\psi_k|H|\psi_j\rangle|^2 \ . \tag{49}$$

Properties of purity of coherence

The important properties of purity of coherence, such as monotonicity under TI operations and convexity, follow from the properties of the function

$$\overline{Q}_2(\rho\|\sigma) \equiv \text{Tr}(\rho^2\sigma^{-1}), \tag{50}$$

if sup$(\rho) \subseteq$ sup(σ), and $\overline{Q}_2(\rho\|\sigma) = \infty$ otherwise. As we will discuss later, the logarithm of this function is the Petz-Rényi relative entropy for $\alpha = 2$.

In particular, this function satisfies the following properties (See [6] for further discussions and proofs of these properties):

- Unitary invariance: It is invariant under any unitary transformation U, i.e. $\overline{Q}_2(U\rho U^\dagger \| U\sigma U^\dagger) = \overline{Q}_2(\rho\|\sigma)$.
- Joint convexity: For any $0 \leq p \leq 1$:

$$p\,\overline{Q}_2(\rho_1\|\sigma_1) + (1-p)\overline{Q}_2(\rho_2\|\sigma_2) \geq \overline{Q}_2\Big([p\rho_1 + (1-p)\rho_2]\Big\|[p\sigma_1 + (1-p)\sigma_2]\Big) \tag{51}$$

- Information-processing inequality: For any completely positive trace-preserving map $\mathcal{E}$,

$$\overline{Q}_2(\mathcal{E}(\rho)\|\mathcal{E}(\sigma)) \leq \overline{Q}_2(\rho\|\sigma) \ . \tag{52}$$

  This follows from the Stinespring dilation theorem, together with unitary invariance and joint convexity.

Using these properties, it can be easily seen that for any time $t \in \mathbb{R}$, the function $B(\rho) \equiv \overline{Q}_2(\rho\|e^{-itH}\rho e^{itH})$ is monotone under any TI operation $\mathcal{E}_{\text{TI}}$:

$$B(\rho) = \overline{Q}_2(\rho\|e^{-itH}\rho e^{itH}) \geq \overline{Q}_2(\mathcal{E}_{\text{TI}}(\rho)\|\mathcal{E}_{\text{TI}}(e^{-itH}\rho e^{itH})) \tag{53}$$

$$= \overline{Q}_2(\mathcal{E}_{\text{TI}}(\rho)\|e^{-itH}\mathcal{E}_{\text{TI}}(\rho)e^{itH}) \tag{54}$$

$$= B(\mathcal{E}_{\text{TI}}(\rho)) \ , \tag{55}$$

where the inequality follows from the information processing inequality for $\overline{Q}_2$, and the second line follows from the fact that $\mathcal{E}_{\text{TI}}$ is a TI operation.

The connection between this function and the purity of coherence follows from the fact that for small Δt,

$$\overline{Q}_2(\rho\|e^{-i\Delta tH}\rho e^{i\Delta tH}) = \text{Tr}(\rho^2(e^{-i\Delta tH}\rho e^{i\Delta tH})^{-1}) \tag{56}$$

$$= \text{Tr}(\rho^2 e^{-i\Delta tH}\rho^{-1}e^{i\Delta tH}) \tag{57}$$

$$= \Big[1+\Delta t^2\text{Tr}(\rho^2 H\rho^{-1}H) - \Delta t^2\text{Tr}(\rho^2H^2\rho^{-1})/2 - \Delta t^2\text{Tr}(\rho^2\rho^{-1}H^2)/2 + \mathcal{O}(\Delta t^4)\Big]$$

$$= 1 + \Delta t^2 P_H(\rho) + \mathcal{O}(\Delta t^4)\ . \tag{58}$$

In other words, $P_H(\rho)$ is 2 time the second derivative of function $\overline{Q}_2(\rho\|e^{-itH}\rho e^{itH})$ with respect to the parameter t, at $t=0$.

Then, it follows from the joint convexity of $\overline{Q}_2$ in Supplementary Eq.(51), and information processing inequality in Supplementary Eq.(52), that function P_H is

- Convex: For any $0\le p\le 1$, and any pair of states ρ_1,ρ_2 :

$$pP_H(\rho_1)+(1-p)P_H(\rho_2) \ge P_H([p\rho_1+(1-p)\rho_2])\ . \tag{59}$$

- Monotone: For any TI operation $\mathcal{E}_{\text{TI}}$

$$P_H(\mathcal{E}_{\text{TI}}(\rho)) \le P_H(\rho). \tag{60}$$

Furthermore, it turns out that the purity of coherence has the following useful properties:

- Additive: For a composite non-interacting system with the total Hamiltonian $H_{\text{tot}} = H_1\otimes I_2 + I_1\otimes H_2$, the purity of coherence is additive for uncorrelated states, i.e. $F_{H_{\text{tot}}}(\rho_1\otimes\rho_2) = P_{H_1}(\rho_1) + P_{H_2}(\rho_2)$. This follows from the fact that $\overline{Q}_2(\rho_1\otimes\rho_2\|\sigma_1\otimes\sigma_2) = \overline{Q}_2(\rho_1\|\sigma_1) + \overline{Q}_2(\rho_2\|\sigma_2)$.

- Faithful: It is non-negative, and is zero if, and only if, state is incoherent, i.e. diagonal in the energy eigenbasis.

  To see this consider the spectral decomposition of state ρ as $\rho=\sum_j p_j|\psi_j\rangle\langle\psi_j|$. Then, we obtain the formula

$$P_H(\rho) = \sum_{j,k}\frac{p_k^2-p_j^2}{p_j}\ |\langle\psi_k|H|\psi_j\rangle|^2 \tag{61a}$$

$$= \sum_{j,k}\frac{p_k-p_j}{p_j}\ (p_k+p_j)|\langle\psi_k|H|\psi_j\rangle|^2, \tag{61b}$$

$$= \sum_{j,k}\frac{(p_j-p_k)^2}{2p_jp_k}(p_j+p_k)|\langle\psi_k|H|\psi_j\rangle|^2\ , \tag{61c}$$

  where to get the third line we have used the fact that $(p_j+p_k)|\langle\psi_k|H|\psi_j\rangle|^2$ is symmetric with respect to k and j. The immediately implies that $P_H(\rho)\ge 0$. Furthermore, since all the terms in the summation are non-negative, the sum will be zero iff all the individual terms are zero. That is $(p_j-p_k)^2|\langle\psi_k|H|\psi_j\rangle|^2 = 0$ for all j,k, or equivalently $(p_j-p_k)\langle\psi_k|H|\psi_j\rangle = 0$. Multiplying both sides in $|\psi_k\rangle\langle\psi_j|$, and summing over j and k this implies $[\rho,H]=0$ and completes the proof.

Connection with Petz-Rényi relative entropy

Similar to QFI, function $P_H(\rho)$ also determines how fast state ρ becomes distinguishable from its time evolved version $e^{-iHt}\rho e^{iHt}$ and is closely related to the Petz-Rényi relative entropies. For $\alpha\in(0,1)\cup(1,2]$ The Petz-Rényi relative entropy for is defined as

$$D_\alpha(\rho\|\sigma) = \frac{1}{\alpha-1}\log\text{Tr}(\rho^\alpha\sigma^{1-\alpha}),\qquad \text{supp}(\rho)\subseteq\text{supp}(\sigma),\text{or }\alpha\in(0,1) \tag{62}$$

$$D_\alpha(\rho\|\sigma) = \infty,\qquad \text{otherwise} \tag{63}$$

Note that the in the special case of $\alpha=2$, we have $D_2(\rho\|\sigma) = \log\overline{Q}_2(\rho\|\sigma)$.

The relative Rényi entropy can be interpreted as a measure of distinguishability of states. In particular, it is non-negative and $D_\alpha(\rho\|\sigma)$ is zero if and only if $\rho=\sigma$. Furthermore, it satisfies information processing inequality for $\alpha\in[0,2]/\{1\}$ [6, 7], that is for any CPTP map $\mathcal{E}$, it holds that $D_\alpha(\rho\|\sigma)\geq D_\alpha(\mathcal{E}(\rho)\|\mathcal{E}(\sigma))$.

It can be easily seen that for small time Δt,

$$D_\alpha(\rho\|e^{-i\Delta tH}\rho e^{i\Delta tH})=\frac{1}{\alpha-1}\Delta t^2[\mathrm{Tr}(\rho^\alpha H\rho^{1-\alpha}H)-\mathrm{Tr}(\rho H^2)]+\mathcal{O}(\Delta t^4)\ . \tag{64}$$

Then, using the arguments we used in the case of the purity of coherence, we can see that all functions in the family

$$\mathrm{Tr}(\rho^\alpha H\rho^{1-\alpha}H)-\mathrm{Tr}(\rho H^2)\ ,\qquad 1<\alpha\leq 2 \tag{65}$$

satisfy all the essential properties of the purity of coherence, such as monotonicity under TI operations, additivity, and convexity.

The reason that in this paper we focus on the case of $\alpha=2$, is that for higher values of α, monotonicity under TI operations, which follows from the monotonicity of Petz-Rényi relative entropy under data processing, does not hold. On the other hand, for lower values of α, as a mixed state ρ converges to a pure state, function $\mathrm{Tr}(\rho^\alpha H\rho^{1-\alpha}H)-\mathrm{Tr}(\rho H^2)$, has slower divergence. Therefore, to see the unreachability of pure coherent states, $\alpha=2$ is the optimal choice.

Stochastic state conversions under TI operations (Proof of Eq.5 in the paper)

Suppose there exists a TI operation which transforms n copies of a system with state ρ_1 and Hamiltonian H_1 to a single copy of a system with state ρ_2 and Hamiltonian H_2, with probability of success $p>0$.

First, assume the transformation is deterministic, i.e. the probability of success p is equal to one. Then, the monotonicity of the purity of coherence under TI operations implies the purity of coherence at the output is less than or equal to the purity of coherence at the input. Using the additivity of purity of coherence, we find the purity of coherence at the input is $n\times P_{H_1}(\rho_1)$. Therefore, we conclude

$$n\geq\frac{P_{H_2}(\rho_2)}{P_{H_1}(\rho_1)}\ . \tag{66}$$

Next, consider the case where the transformation is stochastic, i.e $p\leq 1$. This means that there exists a completely positive TI map $\tilde{\mathcal{E}}$, which is not necessarily trace-preserving, such that

$$\tilde{\mathcal{E}}(\rho_1^{\otimes n})=p\rho_2\ . \tag{67}$$

Then, define

$$\mathcal{F}(\cdot)\equiv|0\rangle\langle 0|\otimes\tilde{\mathcal{E}}(\cdot)+[1-\mathrm{Tr}(\tilde{\mathcal{E}}(\cdot))](|1\rangle\langle 1|\otimes\sigma_{\text{incoh}})\ , \tag{68}$$

where σ_{incoh} is an incoherent state (e.g. the totally mixed state), $\{|0\rangle,|1\rangle\}$ are orthonormal states of a *register* qubit with Hamiltonian zero. In other words, the Hamiltonian of the output is $I\otimes H_2$, where I is the identity operator on the register.

It can be easily shown that map $\mathcal{F}$ is (i) trace-preserving and completely positive, (ii) it is a TI operation, and (iii) implements the transformation

$$\mathcal{F}(\rho_1^{\otimes n})=p|0\rangle\langle 0|\otimes\rho_2+(1-p)(|1\rangle\langle 1|\otimes\sigma_{\text{incoh}})\ . \tag{69}$$

Using definition $P_H(\rho)\equiv\mathrm{Tr}(H\rho^2H\rho^{-1})-\mathrm{Tr}(\rho H^2)$, it can be easily shown that the purity of coherence of this output state is

$$pP_{H_2}(\rho_2)+(1-p)P_{H_2}(\sigma_{\text{incoh}})=p\times P_{H_2}(\rho_2)\ , \tag{70}$$

Since $\mathcal{F}$ is a TI operation, the purity of coherence of its output is less than or equal to the purity of coherence of its input $\rho_1^{\otimes n}$, which is equal to $n\times P_{H_1}(\rho_1)$. We conclude that

$$n\geq p\times\frac{P_{H_2}(\rho_2)}{P_{H_1}(\rho_1)}\ . \tag{71}$$

Thus, to generate a single copy of a pure state ρ_2 which contains coherence, we need $n=\infty$ or $P_{H_1}(\rho_1)=\infty$.

States with infinite purity of coherence

The definition of the purity of coherence immediately implies that for any pure state which is not an eigenstate of Hamiltonian the purity of coherence is infinite. As we saw above, this unboundedness, reflects the fact that given any finite copies of a generic mixed state (with full-rank density operator) it is impossible to create a single copy such pure states using TI operations. More generally,

Lemma 1 For a bounded Hamiltonian H (i.e. $\|H\|_\infty < \infty$) the purity of coherence $P_H(\rho) < \infty$ if and only if $[\Pi_\rho, H] = 0$, where Π_ρ is the projector to the support of ρ. In particular, the purity of coherence is bounded for states with full rank.

Proof. For a bounded Hamiltonian H, $P_H(\rho) = \infty$ if, and only if, the operator $H\rho^2 H$ has support outside the support of ρ. The support of operator $H\rho^2 H$, is equal to the support of operator $H\Pi_\rho H$. Therefore, $P_H(\rho) < \infty$ if, and only if $Q_\rho H \Pi_\rho H Q_\rho = 0$, where $Q_\rho = I - \Pi_\rho$ is the projector to the kernel of ρ. The last equality holds only iff $Q_\rho H \Pi_\rho = 0$, which means $[H, \Pi_\rho] = 0$. We conclude that $P_H(\rho) = \infty$, if and only if $[H, \Pi_\rho] \neq 0$.

□

The following proposition follows immediately from this lemma together with the monotonicity of the purity of coherence under TI operations.

Remark. Let H_{in} and H_{out} be the Hamiltonians of the input and output systems, respectively. Suppose under a TI operation the input state ρ is transformed to the output state σ. Let Π_ρ and Π_σ be the projectors to the supports of ρ and σ, respectively. If $[\Pi_\rho, H_{\text{in}}] = 0$ then $[\Pi_\sigma, H_{\text{out}}] = 0$.

In the following, we present an interpretation and a different proof of this result in terms of the notion of unambiguous state discrimination [8], which clarifies the physical relevance of condition $[H, \Pi_\rho] = 0$.

Recall that two density operators can be unambiguously discriminated with a non-zero probability iff their supports are not identical [8]. The support of state $e^{-iHt}\rho e^{iHt}$ is $e^{-iHt}\Pi_\rho e^{iHt}$, which is equal to Π_ρ for all $t \in \mathbb{R}$, if and only if $[H, \Pi_\rho] = 0$. Therefore, we conclude that there exists $t \in \mathbb{R}$ such that two states $e^{-iHt}\rho e^{iHt}$ and ρ can be unambiguously discriminated with a non-zero probability, iff $[H, \Pi_\rho] \neq 0$.

Next, we note that if the probability of unambiguous discrimination of two states ρ_1 and ρ_2 is zero, then this probability remains zero under any completely positive trace preserving map $\mathcal{E}$, i.e. two states $\sigma_1 = \mathcal{E}(\rho_1)$ and $\sigma_2 = \mathcal{E}(\rho_2)$ will also have the same support. This immediately implies that if the probability of unambiguous discrimination of ρ and $e^{-iHt}\rho e^{iHt}$ is zero, then for any TI operation $\mathcal{E}_{\text{TI}}$, the probability of unambiguous discrimination of the two states $\sigma = \mathcal{E}_{\text{TI}}(\rho)$ and

$$\mathcal{E}_{\text{TI}}(e^{-iHt}\rho e^{iHt}) = e^{-iHt}\mathcal{E}_{\text{TI}}(\rho)e^{iHt} = e^{-iHt}\sigma e^{iHt} \tag{72}$$

should also be zero. We conclude that if $[\Pi_\rho, H] = 0$, and $\sigma = \mathcal{E}_{\text{TI}}(\rho)$ for a TI operation $\mathcal{E}_{\text{TI}}$, then $[\Pi_\sigma, H] = 0$.

Purity of coherence is lower-bounded by Quantum Fisher Information

In this section we show that the purity of coherence is lower-bounded by the Quantum Fisher Information (QFI). That is for any state ρ, and Hamiltonian H, $P_H(\rho) \geq F_H(\rho)$.

For state ρ with the spectral decomposition $\rho = \sum_j p_j |\psi_j\rangle\langle\psi_j|$, the QFI is given by

$$F_H(\rho) = 2\sum_{k,l} \frac{(p_k - p_l)^2}{p_k + p_l} |\langle\psi_k|H|\psi_l\rangle|^2 \ , \tag{73}$$

Recall form Supplementary Eq.61 that the purity of coherence can be rewritten as

$$P_H(\rho) = \sum_{j,k} \frac{p_k^2 - p_j^2}{p_j} \ |\langle\psi_k|H|\psi_j\rangle|^2 = \sum_{k,l} (p_k - p_l)^2 \times \frac{p_k + p_l}{2p_l p_k} |\langle\psi_k|H|\psi_l\rangle|^2 \ . \tag{74}$$

Using the arithmetic-geometric mean inequality applied to p_k and p_l, we have

$$\frac{p_k + p_l}{2p_l p_k} \geq \frac{2}{p_k + p_l} \ . \tag{75}$$

This implies that

$$P_H(\rho) = \sum_{k,l} (p_k - p_l)^2 \frac{(p_k + p_l)}{2p_l p_k} |\langle\psi_k|H|\psi_l\rangle|^2 \geq \sum_{k,l} (p_k - p_l)^2 \frac{2}{p_k + p_l} |\langle\psi_k|H|\psi_l\rangle|^2 = F_H(\rho) \,, \tag{76}$$

where in the last step we have used Supplementary Eq.73 for QFI. Therefore, the inequality $P_H(\rho) \geq F_H(\rho)$ basically follows from arithmetic-geometric mean inequality [24].

Note that the arithmetic-geometric mean inequality holds as equality only if $p_k = p_l$. This means that, if $(p_k - p_l)\langle\psi_k|H|\psi_l\rangle$ is non-zero for some l, k, then $P_H(\rho) > F_H(\rho)$. Equivalently, this means that $P_H(\rho) = F_H(\rho)$, only if

$$(p_k - p_l)\langle\psi_k|H|\psi_l\rangle = 0 \ : \forall k, l \,. \tag{77}$$

Multiplying both sides in $|\psi_k\rangle\langle\psi_l|$ and summing over l, k, this equation is equivalent to

$$\sum_k p_k |\psi_k\rangle\langle\psi_k| H \sum_l |\psi_l\rangle\langle\psi_l| = \sum_k |\psi_k\rangle\langle\psi_k| H \sum_l p_l |\psi_l\rangle\langle\psi_l| \,, \tag{78}$$

which is equivalent to $\rho H = H\rho$. I.e. the equality holds iff ρ is incoherent, in which case both quantities $F_H(\rho)$ and $P_H(\rho)$ are zero.

Purity of coherence for Qubits

Consider a general qubit state ρ with the spectral decomposition

$$\rho = p|\psi\rangle\langle\psi| + (1-p)|\psi^\perp\rangle\langle\psi^\perp| \tag{79}$$

where $\langle\psi^\perp|\psi\rangle = 0$. Then,

$$\rho^{-1} = \frac{1}{p}|\psi\rangle\langle\psi| + \frac{1}{1-p}|\psi^\perp\rangle\langle\psi^\perp| \,, \tag{80}$$

and

$$\rho^2 = p^2|\psi\rangle\langle\psi| + (1-p)^2|\psi^\perp\rangle\langle\psi^\perp|. \tag{81}$$

This implies

$$\mathrm{Tr}(\rho^2 H \rho^{-1} H) = p\mathrm{Tr}(H|\psi\rangle\langle\psi|H|\psi\rangle\langle\psi|) + \frac{p^2}{1-p}\mathrm{Tr}(H|\psi\rangle\langle\psi|H|\psi^\perp\rangle\langle\psi^\perp|) \tag{82}$$

$$+ \frac{(1-p)^2}{p}\mathrm{Tr}(H|\psi^\perp\rangle\langle\psi^\perp|H|\psi\rangle\langle\psi|) + (1-p)\mathrm{Tr}(H|\psi^\perp\rangle\langle\psi^\perp|H|\psi^\perp\rangle\langle\psi^\perp|) \,. \tag{83}$$

Using the fact that

$$\mathrm{Tr}(H|\psi\rangle\langle\psi|H|\psi^\perp\rangle\langle\psi^\perp|) = V_H(\psi) = V_H(\psi^\perp) \,, \tag{84}$$

we find

$$\mathrm{Tr}(\rho^2 H \rho^{-1} H) = \Big(\frac{p^2}{1-p} + \frac{(1-p)^2}{p}\Big) \times V(\psi) + p|\langle\psi|H|\psi\rangle|^2 + (1-p)|\langle\psi^\perp|H|\psi^\perp\rangle|^2 \,. \tag{85}$$

Then, we find

$$P_H(\rho) = \mathrm{Tr}(\rho^2 H \rho^{-1} H) - \mathrm{Tr}(\rho H^2) = \Big(\frac{p^2}{1-p} + \frac{(1-p)^2}{p} - 1\Big) \times V(\psi) \tag{86}$$

$$= \frac{(1-2p)^2}{p(1-p)} \times V(\psi) \,. \tag{87}$$

Next, using the formula for Quantum Fisher information for the family of states $e^{-iHt}\rho e^{iHt}$ with parameter t,

$$F_H(\rho) = 2\sum_{i,j} \frac{(p_i - p_j)^2}{p_i + p_j} |\langle\psi_i|H_S|\psi_j\rangle|^2 \ . \tag{88}$$

where $\sum_i p_i|\psi_i\rangle\langle\psi_i|$ is the spectral decomposition of ρ. Applying this to $\rho = p|\psi\rangle\langle\psi| + (1-p)|\psi^\perp\rangle\langle\psi^\perp|$, we find

$$F_H(\rho) = 4(1-2p)^2 \times V_H(\psi). \tag{89}$$

Therefore,

$$P_H(\rho) = \frac{F_H(\rho)}{4p(1-p)} \ . \tag{90}$$

Finally, note that

$$1 - \text{Tr}(\rho^2) = 1 - [p^2 + (1-p)^2] = 2p - 2p^2 = 2p(1-p). \tag{91}$$

Therefore,

$$P_H(\rho) = \frac{F_H(\rho)}{2[1 - \text{Tr}(\rho^2)]} \ . \tag{92}$$

Purity of coherence for states close to the totally mixed state

Any general state ρ whose trace distance from the totally mixed state is $\|\rho - I/d\|_1 = \epsilon \geq 0$ can be written as

$$\rho = \frac{I}{d} + \epsilon A \ , \tag{93}$$

where A is a Hermitian operator with $\text{Tr}(A) = 0$ and $\|A\|_1 = 1$.
In the following we calculate $P_H(\rho)$ and $F_H(\rho)$ in the limit of $\epsilon \ll 1$.
First, recall that

$$P_H(\rho) = Tr(H\rho^2 H\rho^{-1}) - \text{Tr}(\rho H^2) \ . \tag{94}$$

For $\rho = \frac{I}{d} + \epsilon A$ we find

$$\text{Tr}(\rho H^2) = \frac{1}{d}\text{Tr}(H^2) + \epsilon\text{Tr}(H^2 A) \tag{95}$$

Then,

$$\begin{aligned}
\text{Tr}(H\rho^2 H\rho^{-1}) &= \text{Tr}(H[\frac{I}{d^2} + \frac{2}{d}\epsilon A + \epsilon^2 A^2]H[\frac{I}{d} + \epsilon A]^{-1}) && (96)\\
&= d\text{Tr}(H[\frac{I}{d^2} + \frac{2}{d}\epsilon A + \epsilon^2 A^2]H\frac{1}{I + \epsilon dA}) && (97)\\
&= d\text{Tr}(H[\frac{I}{d^2} + \frac{2}{d}\epsilon A + \epsilon^2 A^2]H[I - \epsilon dA + (\epsilon dA)^2 + \mathcal{O}(\epsilon^3)]) && (98)\\
&= \frac{1}{d}\text{Tr}(H^2) + d\text{Tr}(H[\frac{2}{d}\epsilon A]HI) + d\text{Tr}(H[\frac{I}{d^2}]H[-\epsilon dA]) && (99)\\
&\quad + d\text{Tr}(H[\frac{2}{d}\epsilon A]H[-\epsilon dA]) + d\text{Tr}(H[\epsilon^2 A^2]HI) + d\text{Tr}(H[\frac{I}{d^2}]H[(\epsilon dA)^2]) + \mathcal{O}(\epsilon^3) && (100)\\
&= \frac{1}{d}\text{Tr}(H^2) + \epsilon\text{Tr}(H^2 A) + d2\epsilon^2[\text{Tr}(H^2 A^2) - \text{Tr}(HAHA)] + \mathcal{O}(\epsilon^3) \ . && (101)
\end{aligned}$$

Therefore

$$P_H(\rho) = Tr(H\rho^2 H\rho^{-1}) - \text{Tr}(\rho H^2) = \epsilon^2 2d[\text{Tr}(H^2A^2) - 2\text{Tr}(HAHA)] + \mathcal{O}(\epsilon^3). \tag{102}$$

Next, we calculate Quantum Fisher Information for this state. Recall the formula

$$F_H(\rho) = 2\sum_{i,j} \frac{(p_i - p_j)^2}{p_i + p_j} |\langle\psi_i|H_S|\psi_j\rangle|^2 \ . \tag{103}$$

Let $A = \sum_i a_i|i\rangle\langle i|$ be the spectral decomposition of A. Then,

$$\rho = \frac{I}{d} + \epsilon A = \sum_i (\epsilon a_i + \frac{1}{d})|i\rangle\langle i| \ . \tag{104}$$

This implies

$$F_H(\rho) = 2\sum_{i,j} \frac{\epsilon^2(a_i - a_j)^2}{2/d + \epsilon(a_i + a_j)} |\langle i|H|j\rangle|^2 \ . \tag{105}$$

Expanding this we find

$$F_H(\rho) = d\epsilon^2 \sum_{i,j} \frac{(a_i - a_j)^2}{1 + \epsilon d(a_i + a_j)/2} |\langle i|H|j\rangle|^2 \tag{106}$$

$$= d\epsilon^2 \sum_{i,j} (a_i - a_j)^2 |\langle i|H|j\rangle|^2 + \mathcal{O}(\epsilon^3) \tag{107}$$

$$= d\epsilon^2 \sum_{i,j} (a_i^2 + a_j^2 - 2a_i a_j) |\langle i|H|j\rangle|^2 + \mathcal{O}(\epsilon^3) \tag{108}$$

$$= 2d\epsilon^2 \text{Tr}(A^2H^2) - 2d\epsilon^2 \text{Tr}(HAHA) + \mathcal{O}(\epsilon^3) \tag{109}$$

$$= 2d\epsilon^2 [\text{Tr}(A^2H^2) - \text{Tr}(HAHA)] + \mathcal{O}(\epsilon^3) \ . \tag{110}$$

Comparing this with $P_H(\rho) = \epsilon^2 2d[\text{Tr}(H^2A^2) - 2\text{Tr}(HAHA)] + \mathcal{O}(\epsilon^3)$ we find

$$\frac{P_H(\rho)}{F_H(\rho)} = 1 + \mathcal{O}(\epsilon) \ . \tag{111}$$

Finally, recall that for any pair of states σ_1 and σ_2, it holds that

$$\|\sigma_1 - \sigma_2\|_1 \leq 2\sqrt{1 - \text{Fid}(\sigma_1, \sigma_2)} \ , \tag{112}$$

where $\text{Fid}(\sigma_1, \sigma_2) = \|\sqrt{\sigma_1}\sqrt{\sigma_2}\|_1^2$, and $1 - \text{Fid}(\sigma_1, \sigma_2)$ is called the infidelity of σ_1 and σ_2.

Combining this with Supplementary Eq.111, we conclude that if the infidelity of state ρ and the maximally mixed state I/d is δ, then

$$\frac{P_H(\rho)}{F_H(\rho)} = 1 + \mathcal{O}(\sqrt{\delta}) \ . \tag{113}$$

Supplementary Note 3: Purity of coherence for a mixed state close to a pure state

In this section we find a useful bound on the purity of coherence for mixed states which are close to a pure state. This bound will be used later to study coherence distillation.

Lemma 2 Let $p_{\text{max}} = \|\sigma\|_\infty$ be the largest eigenvalue of σ and $|\Phi\rangle$ be the corresponding eigenvector. Then,

$$P_H(\sigma) \geq \mathrm{V}_H(\Phi) \times (\frac{p_{\text{max}}^2}{1-p_{\text{max}}} - 1) . \tag{114}$$

Proof. Recall that for any state ρ with the spectral decomposition $\rho = \sum_j q_j |\phi_j\rangle\langle\phi_j|$, purity of coherence is given by

$$P_H(\rho) = \sum_{j,k} \frac{q_k^2 - q_j^2}{q_j} |\langle\phi_k|H|\phi_j\rangle|^2 . \tag{115}$$

Consider the spectral decomposition of σ, i.e.

$$\sigma = p_{\text{max}} |\Phi\rangle\langle\Phi| + \sum_j p_j |\psi_j^\perp\rangle\langle\psi_j^\perp| , \tag{116}$$

where $p_{\text{max}} = \|\sigma\|_\infty$ is the largest eigenvalue of σ, $|\Phi\rangle$ is the corresponding eigenvector, and $\{p_j\}_j$ are the rest of the eigenvalues, and $\{|\psi_j^\perp\rangle\}$ are the corresponding eigenvectors. Then, using the general formula for purity of coherence, in Supplementary Eq.(115), and using the fact that for any pair of j and k the sum of two terms

$$(\frac{p_k^2 - p_j^2}{p_j} + \frac{p_j^2 - p_k^2}{p_k})|\langle\psi_k^\perp|H|\psi_j^\perp\rangle|^2 = \frac{(p_j - p_k)^2}{2p_j p_k}(p_j + p_k)|\langle\psi_k^\perp|H|\psi_j^\perp\rangle|^2 \geq 0 , \tag{117}$$

is non-negative, we find

$$P_H(\sigma) \geq \sum_j (\frac{p_{\text{max}}^2 - p_j^2}{p_j} + \frac{p_j^2 - p_{\text{max}}^2}{p_{\text{max}}}) |\langle\Phi|H|\psi_j^\perp\rangle|^2 , \tag{118}$$

where in the summation we have dropped all the terms which do not involve $|\Phi\rangle$. Then, we find

$$\begin{aligned}
P_H(\sigma) &\geq \sum_j (\frac{p_{\text{max}}^2 - p_j^2}{p_j} + \frac{p_j^2 - p_{\text{max}}^2}{p_{\text{max}}}) |\langle\Phi|H|\psi_j^\perp\rangle|^2 && (119)\\
&\geq \sum_j (\frac{p_{\text{max}}^2}{p_j} - [p_{\text{max}} + p_j]) |\langle\Phi|H|\psi_j^\perp\rangle|^2 && (120)\\
&\geq \sum_j (\frac{p_{\text{max}}^2}{p_j} - 1) |\langle\Phi|H|\psi_j^\perp\rangle|^2 && (121)\\
&\geq (\frac{p_{\text{max}}^2}{1-p_{\text{max}}} - 1) \sum_j |\langle\Phi|H|\psi_j^\perp\rangle|^2 && (122)\\
&= (\frac{p_{\text{max}}^2}{1-p_{\text{max}}} - 1) V_H(\Phi) , && (123)
\end{aligned}$$

where to get the third inequality we have used the fact that $p_{\text{max}} + p_j \leq 1$, to get the fourth inequality we have used the fact that $p_j \leq 1 - p_{\text{max}}$, and to get the last equality we have used

$$\begin{aligned}
\sum_j |\langle\Phi|H|\psi_j^\perp\rangle|^2 &= \langle\Phi|H(\sum_j |\psi_j^\perp\rangle\langle\psi_j^\perp| + |\Phi\rangle\langle\Phi|)H|\Phi\rangle - \langle\Phi|H(|\Phi\rangle\langle\Phi|)H|\Phi\rangle && (124)\\
&= \langle\Phi|H^2|\Phi\rangle - \langle\Phi|H|\Phi\rangle^2 = V_H(\Phi) . && (125)
\end{aligned}$$

This completes the proof of lemma. □

This lemma has the following corollary.

Corollary 1 Let $\delta \equiv 1 - \langle\Psi|\sigma|\Psi\rangle$ be the infidelity of pure state Ψ and state σ. Let p_{max} be the largest eigenvalue of σ, and Φ be the corresponding eigenvector. Then, the fidelity of Ψ and Φ is lower bounded by $|\langle\Psi|\Phi\rangle|^2 \geq 1 - 2\delta$, and p_{max} satisfies $p_{\text{max}} \geq 1 - \delta$. Furthermore,

$$P_H(\sigma) \geq V_H(\Phi) \times (\frac{(1-\delta)^2}{\delta} - 1), \tag{126}$$

$$F_H(\sigma) \geq V_H(\Phi) \times 4(1-2\delta)^2 \ . \tag{127}$$

Assuming the infideliy $\delta \leq 1/2$, this means that

$$P_H(\sigma) \geq V_H(\Phi) \times (\frac{1}{4\delta} - 1) \ , \tag{128}$$

$$\tag{129}$$

Proof. Let

$$\sigma = p_{\text{max}}|\Phi\rangle\langle\Phi| + \sum_j p_j|\psi_j^\perp\rangle\langle\psi_j^\perp| \ , \tag{130}$$

be the eigen-decomposition of σ, where p_{max} is the largest eigenvalue and $|\Phi\rangle$ is the corresponding eigenvector. The fact that $\langle\Psi|\sigma|\Psi\rangle = 1-\delta$ implies that

$$1-\delta = \langle\Psi|\sigma|\Psi\rangle = p_{\text{max}}|\langle\Psi|\Phi\rangle|^2 + \sum_j p_j|\langle\Psi|\psi_j^\perp\rangle|^2 \ . \tag{131}$$

Since $|\langle\Psi|\Phi\rangle|^2 + \sum_j |\langle\Psi|\psi_j^\perp\rangle|^2 = 1$, we can interpret this sum as the average of eigenvalues of σ, weighted by the probability distribution defined by $\{|\langle\Psi|\Phi\rangle|^2, |\langle\Psi|\psi_j^\perp\rangle|^2\}$. This average is less than or equal to the maximum eigenvalue, p_{max}, i.e.

$$1-\delta = p_{\text{max}}|\langle\Psi|\Phi\rangle|^2 + \sum_j p_j|\langle\Psi|\psi_j^\perp\rangle|^2 \leq p_{\text{max}} \ , \tag{132}$$

as claimed in the statement of the corollary.

Next, note that

$$\sum_j p_j|\langle\Psi|\psi_j^\perp\rangle|^2 \leq \sum_j p_j \times \sum_k |\langle\Psi|\psi_k^\perp\rangle|^2 = (1-p_{\text{max}})(1-|\langle\Psi|\Phi\rangle|^2) \ , \tag{133}$$

where we have used the facts that $p_{\text{max}} + \sum_j p_j = \text{Tr}(\sigma) = 1$, and the fact that $|\langle\Psi|\Phi\rangle|^2 + \sum_j |\langle\Psi|\psi_j^\perp\rangle|^2 = 1$, because $\{|\Phi\rangle, |\psi_j^\perp\rangle\}$ is an orthonormal basis.

Therefore,

$$1-\delta = \langle\Psi|\sigma|\Psi\rangle \tag{134}$$

$$= p_{\text{max}}|\langle\Psi|\Phi\rangle|^2 + \sum_j p_j|\langle\Psi|\psi_j^\perp\rangle|^2 \tag{135}$$

$$\leq p_{\text{max}}|\langle\Psi|\Phi\rangle|^2 + (1-p_{\text{max}})(1-|\langle\Psi|\Phi\rangle|^2) \tag{136}$$

$$\leq p_{\text{max}}|\langle\Psi|\Phi\rangle|^2 + \delta \times (1-|\langle\Psi|\Phi\rangle|^2) \ , \tag{137}$$

where to get the third line we have used Supplementary Eq.(133) and to get the fourth line we have used E.(132), which implies $\delta \geq 1 - p_{\text{max}}$.

This implies

$$|\langle\Psi|\Phi\rangle|^2 \geq \frac{1-2\delta}{p_{\text{max}}-\delta} \geq 1-2\delta \;, \tag{138}$$

as claimed in the statement of the corollary.

Next, to prove the lower bound on the purity of coherence in Supplementary Eq.126, we use the lower bound in lemma 2 together with Supplementary Eq. 132. This implies

$$P_H(\sigma) \geq V_H(\Phi) \times [\frac{p_{\text{max}}^2}{1-p_{\text{max}}} - 1] \tag{139a}$$

$$\geq V_H(\Phi) \times [\frac{(1-\delta)^2}{\delta} - 1] \;. \tag{139b}$$

This proves Supplementary Eq.126 in the corollary 1.

Next, we prove Supplementary Eq.127, i.e. the lower bound on QFI. Using the spectral decomposition $\sigma = p_{\text{max}}|\Phi\rangle\langle\Phi| + \sum_j p_j|\psi_j^\perp\rangle\langle\psi_j^\perp|$, the formula for QFI in Supplementary Eq.73 can be rewritten as

$$F_H(\sigma) = 4\sum_j \frac{(p_{\text{max}}-p_j)^2}{p_{\text{max}}+p_j} \; |\langle\Phi|H|\psi_j^\perp\rangle|^2 + 2\sum_{j,k} \frac{(p_k-p_j)^2}{p_k+p_j} \; |\langle\psi_k^\perp|H|\psi_j^\perp\rangle|^2 \tag{140a}$$

$$\geq 4\sum_j \frac{(p_{\text{max}}-p_j)^2}{p_{\text{max}}+p_j} \; |\langle\Phi|H|\psi_j^\perp\rangle|^2 \tag{140b}$$

$$\geq 4\sum_j (p_{\text{max}}-p_j)^2 \; |\langle\Phi|H|\psi_j^\perp\rangle|^2 \;, \tag{140c}$$

where to get the second line we ignore all the terms which do not contain p_{max}, and to get the third line we have used $p_j + p_{\text{max}} \leq 1$. Also, using $p_j + p_{\text{max}} \leq 1$, or equivalently, $p_j \leq 1 - p_{\text{max}}$, we have

$$p_{\text{max}} - p_j \geq 2p_{\text{max}} - 1 \geq 2(1-\delta) - 1 = 1 - 2\delta \;, \tag{141}$$

where the second inequality follows from Supplementary Eq.132.

Combining this with Supplementary Eq.140, we find

$$F_H(\sigma) \geq 4\sum_j (p_{\text{max}}-p_j)^2 \; |\langle\Phi|H|\psi_j^\perp\rangle|^2 \tag{142}$$

$$\geq 4(1-2\delta)^2 \sum_j |\langle\Phi|H|\psi_j^\perp\rangle|^2 \tag{143}$$

$$= 4(1-2\delta)^2 \times V_H(\Phi) \;. \tag{144}$$

□

Supplementary Note 4: QFI and purity of coherence in the iid regime (Proof of Eq. 10 and Eq. 11 in the paper)

Theorem 2 Consider m non-interacting systems, each with Hamiltonian H, and with the total Hamiltonian $H_{\text{tot}} = \sum_{i=1}^m H^{(i)}$. Let σ_m be their joint state. Suppose the fidelity of this state with state $|\phi\rangle^{\otimes m}$ is $\langle\phi|^{\otimes m}\sigma_m|\phi\rangle^{\otimes m} = 1-\epsilon_m$, where $|\phi\rangle$ is a pure state with positive energy variance, i.e. $V_H(\phi) > 0$. Then, for sufficiently large m, e.g. $m \geq 70\frac{|\langle\phi|H^3|\phi\rangle|^2}{V_H^3(\phi)}$ and sufficiently small ϵ_m, e.g. $\epsilon_m \leq 10^{-3}$, the QFI of state σ_m and its purity of coherence, relative to the total Hamiltonian H_{tot} are lower bounded by

$$F_{H_{\text{tot}}}(\sigma_m) \geq 4 \times c \times m \times F_H(\phi) \; , \tag{145a}$$

$$P_{H_{\text{tot}}}(\sigma_m) \geq c \times m \times F_H(\phi) \times \frac{1}{\epsilon_m} \; , \tag{145b}$$

where c is a positive constant, e.g. $c = 10^{-2}$.

Proof. We use corollary 1. According to this result, the fact that $\langle\phi|^{\otimes m}\sigma_m|\phi\rangle^{\otimes m} = 1-\epsilon_m$ implies that

$$|\langle\phi^{\otimes m}|\Theta_m\rangle|^2 \geq 1-2\epsilon_m \; , \tag{146}$$

where $|\Theta_m\rangle$ is the eigenvector of σ_m with the largest eigenvalue. Furthermore, the corollary implies that

$$P_{H_{\text{tot}}}(\sigma_m) \geq V_{H_{\text{tot}}}(|\Theta_m\rangle) \times (\frac{(1-\epsilon_m)^2}{\epsilon_m} - 1) \; , \tag{147}$$

Assuming $\epsilon_m \leq 10^{-3}$, this implies

$$P_{H_{\text{tot}}}(\sigma_m) \geq V_{H_{\text{tot}}}(|\Theta_m\rangle) \times \frac{1}{\epsilon_m} \times (0.997) \; . \tag{148}$$

Next, we use the following lemma which is a lower bound on the energy variance of pure states which are close to an iid pure state (This lemma is proven in Sec.3).

Lemma 3 Consider m copies of a system with Hamiltonian H and pure state ϕ with positive energy variance, i.e. $V_H(\phi) > 0$. Assume m is sufficiently large, e.g. $m \geq 70\frac{|\langle\phi|H^3|\phi\rangle|^2}{V_H^3(\phi)}$. Consider state $|\Theta_m\rangle$ whose fidelity with state $|\phi\rangle^{\otimes m}$ satisfies $|\langle\Theta_m|\phi\rangle^{\otimes m}|^2 \geq 1 - 2.5\times 10^{-3}$. Then, the energy variance of $|\Theta_m\rangle$ is lower bounded by

$$V_{H_{\text{tot}}}(|\Theta_m\rangle) \geq C \times m \times V_H(\phi) \; , \tag{149}$$

where C is a positive constant, e.g. $C = 0.05$.

To apply this lemma, we first note that for $\epsilon_m \leq 10^{-3}$,

$$|\langle\phi^{\otimes m}|\Theta_m\rangle|^2 \geq 1-2\epsilon_m \geq 1-2\times 10^{-3} \geq 1-2.5\times 10^{-3} \; , \tag{150}$$

where we have used Supplementary Eq.146. Therefore, if $m \geq 70\frac{|\langle\phi|H^3|\phi\rangle|^2}{V_H^3(\phi)}$, then we can apply the lemma, which implies

$$V_{H_{\text{tot}}}(|\Theta_m\rangle) \geq C \times m \times V_H(\phi) \; , \tag{151}$$

where C is a positive constant, e.g. $C = 0.05$. Putting this into Supplementary Eq.148, we find

$$P_{H_{\text{tot}}}(\sigma_m) \geq V_{H_{\text{tot}}}(|\Theta_m\rangle) \times \frac{1}{\epsilon_m} \times 0.997 \tag{152}$$

$$\geq C \times m \times V_H(\phi) \times \frac{1}{\epsilon_m} \times 0.997 \tag{153}$$

$$\geq \frac{C \times 0.997}{4} \times \frac{m\, F_H(\phi)}{\epsilon_m} \tag{154}$$

$$\geq c \times \frac{m\, F_H(\phi)}{\epsilon_m} , \tag{155}$$

where $c \geq 10^{-2}$. Here, to get the third line we have used $F_H(\phi) = 4V_H(\phi)$. This proves the lower bound $P_{H_{\text{tot}}}(\sigma_m) \geq 10^{-2} \times \frac{m\, F_H(\phi)}{\epsilon_m}$, stated in theorem 2. The lower bound on QFI in this theorem can also be proven in a similar way by combining lemma 3 above and the lower bound on fidelity in corollary 1. In particular, using this corollary we have

$$F_{H_{\text{tot}}}(\sigma_m) \geq V_{H_{\text{tot}}}(|\Theta_m\rangle) \times 4(1 - 2\epsilon_m)^2 , \tag{156}$$

which together with Supplementary Eq.149 implies

$$F_{H_{\text{tot}}}(\sigma_m) \geq C \times m \times V_H(\phi) \times 4(1 - 2\epsilon_m)^2 \tag{157}$$

$$= C \times m \times F_H(\phi) \times (1 - 2\epsilon_m)^2 \tag{158}$$

$$\geq 4c \times m \times F_H(\phi) , \tag{159}$$

where $c \geq 10^{-2}$ (Note that $\epsilon_m \leq 10^{-3}$, and therefore $(1 - 2\epsilon_m)^2 \geq 0.996$, and $C \geq 0.05$) . Therefore, to complete the proof of theorem 2, we only need to prove lemma 3.

Proof of lemma 3 (A lower bound on the energy variance of pure states which are close to an iid pure state)

Let

$$H_{\text{tot}} = \sum_{i=1}^{m} H^{(i)} = \sum_{E \in \text{spec}(H_{\text{tot}})} E\, \Pi_E \tag{160}$$

be the spectral decomposition of Hamiltonian H_{tot}, where spec(H_{tot}) is the set of eigenvalues, and Π_E is the projector to the subspace with eigenvalue E. Let

$$p_m(E) = \langle\phi|^{\otimes m} \Pi_E |\phi\rangle^{\otimes m} , \tag{161}$$

$$q_m(E) = \langle\Theta_m|\Pi_E|\Theta_m\rangle , \tag{162}$$

be, respectively, the energy distributions of states $|\phi\rangle^{\otimes m}$ and $|\Theta_m\rangle$, relative to Hamiltonian H_{tot}.

Note that p_m, i.e. the energy distribution for state $|\phi\rangle^{\otimes m}$, corresponds to the distribution of sum of m independent and identically distributed random variables, each with non-zero variance $V_H(\phi)$. Therefore, the variance of the distribution p_m is $m \times V_H(\phi)$. On the other hand, the variance of distribution q_m is equal to $V_{H_{\text{tot}}}(|\Theta_m\rangle)$. Next, we argue that the assumption $|\langle\Theta_m|\phi\rangle^{\otimes m}|^2 \geq 1 - 2.5 \times 10^{-3}$ in the statement of lemma implies an upper bound on the total variation distance of p_m and q_m, and then use this to find a lower bound on the variance of distribution q_m, or equivalently, a lower bound on $V_{H_{\text{tot}}}(|\Theta_m\rangle)$.

The total variation distance between p_m and q_m is upper bounded by

$$d_{\text{TV}}(p_m, q_m) \equiv \frac{1}{2} \sum_{E \in \text{spec}(H_{\text{tot}})} |p_m(E) - q_m(E)| \tag{163}$$

$$\leq \frac{1}{2} \left\| |\phi\rangle\langle\phi|^{\otimes m} - |\Theta_m\rangle\langle\Theta_m| \right\|_1 \tag{164}$$

$$= \sqrt{1 - |\langle\Theta_m|\phi\rangle^{\otimes m}|^2} , \tag{165}$$

where to get the second line we have used the monotonicity of l_1-norm under CPTP maps, and the fact that measurement in the energy basis is a CPTP map. The equality in the third line holds for any general pair of normalized pure states. This implies that

if $|\langle\Theta_m|\phi\rangle^{\otimes m}|^2 \geq 1-(0.05)^2 = 1-2.5\times 10^{-3}$, then

$$d_{\rm TV}(p_m, q_m) \leq 0.05\ . \tag{166}$$

Next, we use the following lemma proven at the end of this section, using Berry-Esséen's theorem.

Lemma 4 Let $X_1,\cdots,X_M$ be M independent identically distributed random variables, each with variance $\sigma_X^2>0$ and bounded third moment $\xi=\mathbb{E}(X^3)<\infty$. Let $Z_M=\frac{1}{\sqrt{M}}\sum_{i=1}^M X_i$. Suppose M is sufficiently large, e.g. $M\geq \frac{70\times|\xi|^2}{\sigma_X^6}$. Then, any random variable Y_M whose total variation distance from Z_M is sufficiently small, e.g. $d_{\rm TV}(Z_M,Y_M)\leq 0.05$, has variance $\sigma^2_{Y_M}$ which is lower bounded by

$$\sigma^2_{Y_M} \geq C\times \sigma_X^2, \tag{167}$$

where C is a positive constant, e.g. $C=0.05$.

To apply the lemma, we assume X is the random variable which takes values in the set of eigenvalues of H, with the distribution defined by the weight of state $|\phi\rangle$ in the energy eigen-subspaces of H. This means that the distribution of the random variable $\tilde{Z}_m=\sum_{i=1}^m X_i=\sqrt{m}Z_m$ is given by p_m and its variance is $m\times V_H(\phi)$.

Now suppose the energy distribution of state $|\Theta_m\rangle$ is described by the random variable $\tilde{Y}_m$, which has distribution q_m. This means that its variance is equal to

$$\sigma^2_{\tilde{Y}_m} = V_{H_{\rm tot}}(|\Theta_m\rangle)\ . \tag{168}$$

Define

$$Y_m \equiv \frac{\tilde{Y}_m}{\sqrt{m}}\ . \tag{169}$$

Lemma 4 implies that if $d_{\rm TV}(p_m,q_m)\leq 0.05$, then

$$\sigma^2_{Y_m} \geq C\sigma_X^2 = CV_H(\phi)\ , \tag{170}$$

which in turn implies

$$V_{H_{\rm tot}}(|\Theta_m\rangle) = \sigma^2_{\tilde{Y}_m} = m\times\sigma^2_{Y_m} \geq Cm\sigma_X^2 = CmV_H(\phi)\ . \tag{171}$$

This proves lemma 3. Therefore, to complete the proof of lemma 3 and theorem 2, we only need to prove lemma 4.

□

Proof of lemma 4

Without loss of generality we assume the expectation of the random variable X is zero, i.e. $\mathbb{E}(X)=0$ (Otherwise, we can always add a constant to the random variable and make its expectation zero).

Let p_M and q_M be, respectively, the probability distributions associated to the random variables $Z_M=\frac{1}{\sqrt{M}}\sum_{i=1}^M X_i$ and Y_M. To simplify the notation, we assume they have discrete supports. Suppose their total variation distance is δ, i.e.

$$\frac{1}{2}\sum_y |p_M(y)-q_M(y)| = \delta\ . \tag{172}$$

It follows that for any set S,

$$\sum_{y\in S} q_M(y) \geq \sum_{y\in S} p_M(y) - \delta\ . \tag{173}$$

In particular, for both sets $S_+ \equiv \{y : y > \sigma_X\}$ and $S_- \equiv \{y : y < -\sigma_X\}$, we have

$$\sum_{y\in S_+} q_M(y) \geq \sum_{y\in S_+} p_M(y) - \delta \,, \tag{174a}$$

$$\sum_{y\in S_-} q_M(y) \geq \sum_{y\in S_-} p_M(y) - \delta \,. \tag{174b}$$

Let

$$\mu_{Y_M} = \mathbb{E}(Y_M) = \sum_y q_M(y) y \,, \tag{175}$$

be the expectation of the random variable Y_M. Then, the variance of this random variable is

$$\sigma^2_{Y_M} = \sum_y q_M(y)(y-\mu_{Y_M})^2 \tag{176}$$

$$\geq \sum_{y\in S_+} q_M(y)(y-\mu_{Y_M})^2 + \sum_{y\in S_-} q_M(y)(y-\mu_{Y_M})^2 \,, \tag{177}$$

where the bound follows from the fact that all the terms in the summation are non-negative. Given that the sets $S_+ = \{y : y > \sigma_X\}$ and $S_- = \{y : y < -\sigma_X\}$ are separated by, at least, $2\times\sigma_X$, we can easily see that for any value of μ_{Y_M}, at least, one of the followings holds:

$$\forall y \in S_+ :\ |y-\mu_{Y_M}| > \sigma_X ,$$

or

$$\forall y \in S_- :\ |y-\mu_{Y_M}| > \sigma_X \,.$$

Therefore,

$$\sigma^2_{Y_M} \geq \sum_{y\in S_+} q_M(y)(y-\mu_{Y_M})^2 + \sum_{y\in S_-} q_M(y)(y-\mu_{Y_M})^2 \tag{178}$$

$$\geq \sigma_X^2 \times \min\Big\{ \sum_{y\in S_+} q_M(y) \,,\, \sum_{y\in S_-} q_M(y) \Big\} \,. \tag{179}$$

Combining this with Eqs.174 we find

$$\sigma^2_{Y_M} \geq \sigma_X^2 \times \min\Big\{ \sum_{y\in S_+} p_M(y) \,,\, \sum_{y\in S_-} p_M(y) \Big\} - \sigma_X^2 \times \delta \,. \tag{180}$$

Next, we find a lower bound on $\sum_{y\in S_+} p_M(y)$, and $\sum_{y\in S_-} p_M(y)$. Recall that p_M is the distribution of the random variable $Z_M = \frac{1}{\sqrt{M}} \sum_{i=1}^M X_i$. For sufficiently large M, this distribution converges to the Gaussian distribution, and we can use Berry-Esséen's theorem to find lower bounds on the tails of this distribution.

Berry-Esséen's theorem [9, 10]: Let $X_1, \cdots X_M$ be independent and identically distributed random variables, with means 0, i.e. $\mathbb{E}(X_i) = 0$ and variance $\sigma_X^2 = \mathbb{E}(X_i^2) > 0$, and finite third moment $\xi = \mathbb{E}(X_i^3) < \infty$. Let $W_M = \frac{1}{\sigma_X\sqrt{M}} \sum_{i=1}^M X_i$. Then, for any $a \in \mathbb{R}$, the probability that $W_M < a$, denoted by $\mathbf{P}(W_M < a)$, satisfies

$$\Big|\mathbf{P}(W_M < a) - \mathbf{P}(N < a)\Big| < c \frac{|\xi|}{\sigma_X^3 \sqrt{M}} \,, \tag{181}$$

where N is the random variable with the standard Normal distribution, i.e. with mean zero and variance one, $\mathbf{P}(N < a)$ is the corresponding cumulative distribution, and c is an order one positive constant (E.g. we can choose $c = 1/2$).

Recall that p_M is the distribution associated to the random variable

$$Z_M = \frac{1}{\sqrt{M}} \sum_{i=1}^{M} X_i = \sigma_X \times W_M \ , \tag{182}$$

which means

$$\sum_{y\in S_-} p_M(y) = \sum_{y<-\sigma_X} p_M(y) = \mathbf{P}(Z_M < -\sigma_X) = \mathbf{P}(W_M < -1) \tag{183}$$

and

$$\sum_{y\in S_+} p_M(y) = \sum_{y>\sigma_X} p_M(y) = \mathbf{P}(Z_M > \sigma_X) = \mathbf{P}(W_M > 1) \ . \tag{184}$$

Then, applying Berry-Esséen theorem, we find

$$\Big|\sum_{y\in S_-} p_M(y) - \mathbf{P}(N < -1)\Big| = \Big|\Big(\sum_{y<-\sigma_X} p_M(y)\Big) - \mathbf{P}(N < -1)\Big| < c\frac{|\xi|}{\sigma_X^3\sqrt{M}} \ , \tag{185}$$

and

$$\Big|\sum_{y\in S_+} p_M(y) - \mathbf{P}(N > 1)\Big| = \Big|\Big(\sum_{y>\sigma_X} p_M(y)\Big) - \mathbf{P}(N > 1)\Big| < c\frac{|\xi|}{\sigma^3\sqrt{M}} \ . \tag{186}$$

Given that for the normal distribution N with mean zero and variance one, $P(N > 1) = P(N < -1) > 0.16$, we find

$$\sum_{y\in S_-} p_M(y) = \sum_{y<-\sigma_X} p_M(y) \geq 0.16 - c\frac{|\xi|}{\sigma_X^3\sqrt{M}} \ , \tag{187a}$$

$$\sum_{y\in S_+} p_M(y) = \sum_{y>\sigma_X} p_M(y) \geq 0.16 - c\frac{|\xi|}{\sigma_X^3\sqrt{M}} \ . \tag{187b}$$

Now assume M is sufficiently large, e.g.

$$M \geq \frac{70 \times |\xi|^2}{\sigma_X^6} \ . \tag{188}$$

Since $c = 1/2$, this guarantees that $c \times \frac{|\xi|}{\sigma_X^3\sqrt{M}} \leq 0.06$. Putting this in Supplementary Eq.(187), we find

$$\sum_{y\in S_-} p_M(y) = \sum_{y<-\sigma_X} p_M(y) \geq 0.1 \quad , \text{and} \quad \sum_{y\in S_+} p_M(y) = \sum_{y>\sigma_X} p_M(y) \geq 0.1 \ . \tag{189}$$

Putting this back into Supplementary Eq.(180), we find

$$\sigma_{Y_M}^2 \geq \sigma_X^2 \times \min\Big\{\sum_{y\in S_+} p_M(y) \ , \sum_{y\in S_-} p_M(y)\Big\} - \sigma_X^2\delta \tag{190}$$

$$\geq \sigma_X^2 \times 0.1 \ - \sigma_X^2\delta \ . \tag{191}$$

Assuming the total variation distance $\delta = \frac{1}{2}\sum_y |p_M(y) - q_M(y)| \leq 0.05$, we find

$$\sigma_{Y_M}^2 \geq 0.05 \times \sigma_X^2. \tag{192}$$

This proves lemma 4, and completes the proof of lemma 3 and theorem 2.

Supplementary Note 5: Extension of the main theorem: Finite helper systems do not help

It turns out that our no-go theorem on coherence distillation can be extended to the case where one is allowed to use a finite *helper* system at the input to implement the transformation

$$\rho^{\otimes n} \otimes \chi \xrightarrow{TI} \approx \psi^{\otimes \lceil Rn \rceil}, \tag{193}$$

where χ is the state of the helper system, in a finite-dimensional Hilbert space with a bounded Hamiltonian. The helper system can be in a pure state, in which case the purity of coherence of the input can be ∞, even for finite n. Therefore, in this case it is not clear that how we can put a restriction on the output based on the purity of coherence of the input. Nevertheless, we can overcome this issue, and prove an extension of our no-go theorem, which implies distillable coherence remains zero for states with bounded purity of coherence, even if one allows a finite helper system at the input. This result follows from the following lemma together with an argument similar to the one which proved our no-go theorem.

Lemma 5 Suppose there exists a TI operation $\mathcal{E}_n$ which transforms n copies of system with state ρ and Hamiltonian H and a helper system in state χ and Hamiltonian H_{help}, to m copies of a system with Hamiltonian H and state ψ with error ϵ_n in trace distance, such that

$$\left\| \mathcal{E}_n(\rho^{\otimes n} \otimes \chi) - \psi^{\otimes m} \right\|_1 \leq \epsilon_n \ . \tag{194}$$

Then, there exists a pure state $|\Theta_n\rangle$ (namely the eigenstate of $\mathcal{E}_n(\rho^{\otimes n} \otimes \chi)$ with the largest eigenvalue) whose overlap with the desired state $\psi^{\otimes m}$ is

$$|\langle \Theta_n|\psi\rangle^{\otimes m}|^2 \geq 1 - 4\epsilon_n, \tag{195}$$

and satisfies

$$\epsilon_n P_H(\rho) + 2(d_\chi - 1)\ \frac{1}{n} V_{H_{\text{help}}}(\chi) \geq \frac{1}{n} V_{H_{\text{tot}}}(|\Theta_n\rangle) \times ((1-\epsilon_n)^2 - \epsilon_n)\ , \tag{196}$$

where d_χ is the dimension of the Hilbert space of the helper system, and $H_{\text{tot}} = \sum_{i=1}^m H^{(i)}$ is the sum of the Hamiltonians of the output systems.

Suppose in the limit n goes to ∞, error ϵ_n goes to zero. If $P_H(\rho)$ is bounded, then the left-hand side of the above bound vanishes, which implies

$$\lim_{n\to\infty} \frac{1}{n} V_{H_{\text{tot}}}(|\Theta_n\rangle) = 0\ . \tag{197}$$

Recall that $|\Theta_n\rangle$ is the eigenvector of the output state $\mathcal{E}_n(\rho^{\otimes n} \otimes \chi)$. But, applying lemma 3 we know that for sufficiently large m and sufficiently small ϵ_m,

$$V_{H_{\text{tot}}}(|\Theta_n\rangle) \geq C \times m \times V_H(\psi)\ , \tag{198}$$

where C is a positive constant, e.g. $C = 0.05$. Combining this with Supplementary Eq.197, we conclude that

$$V_H(\psi) \times \lim_{n\to\infty} \frac{m}{n} = 0\ . \tag{199}$$

Therefore, assuming $P_H(\rho)$ is bounded and $V_H(\psi) > 0$, then to have a vanishing error $\epsilon_n \to 0$, we also need to have a vanishing rate. In conclusion, the distillable coherence of states with finite purity of coherence remains zero, even at the presence of finite-dimensional helper systems.

Proof of lemma 5

In general, at the presence of the helper state, the purity of coherence of the input can be ∞ for a finite n, in which case we cannot put any constraint on the output based on its purity of coherence. To rectify this issue we use the following trick, which can be used more generally when one deals with the purity of coherence for pure states: assume instead of using the helper state in the pure state χ, we use τ_χ, a noisy version of χ obtained by mixing χ with the totally mixed state, with a ratio such that

the trace distance between χ and τ_χ is exactly ϵ_n. Now suppose in the process $\rho^{\otimes n}\otimes\chi \xrightarrow{\text{TI}} \psi^{\otimes m}$, we use τ_χ instead of χ. Then, we introduce an additional error in the process. Using the fact that the trace distance satisfies the triangle inequality, and is non-increasing under CPTP maps, this additional error can be bounded by ϵ_n. Therefore, the total error at the output will be bounded by $2\epsilon_n$. To summarize, if

$$\left\|\mathcal{E}_n(\rho^{\otimes n}\otimes\chi)-\psi^{\otimes m}\right\|_1\leq\epsilon_n\ , \tag{200}$$

then,

$$\left\|\mathcal{E}_n(\rho^{\otimes n}\otimes\tau_\chi)-\psi^{\otimes m}\right\|_1\leq 2\epsilon_n\ . \tag{201}$$

In this transformation, the purity of coherence for the input is $nP_H(\rho)+P_{H_\text{help}}(\tau_\chi)$. Later, we show that $P_{H_\text{help}}(\tau_\chi)$ is upper bounded by

$$P_{H_\text{help}}(\tau_\chi)\leq\frac{2(d_\chi-1)}{\epsilon_n}\,V_{H_\text{help}}(\chi)\ , \tag{202}$$

where d_χ is the dimension of the Hilbert space of χ. Therefore, the total purity of coherence for the input $\rho^{\otimes n}\otimes\tau_\chi$ is upper bounded by

$$nP_H(\rho)+\frac{2(d_\chi-1)}{\epsilon_n}\,V_{H_\text{help}}(\chi)\ . \tag{203}$$

Next, we focus on the purity of coherence of the output, and use corollary 1, which provides a lower bound on the purity of coherence for mixed states close to pure states. Let $\sigma=\mathcal{E}_n(\rho^{\otimes n}\otimes\tau_\chi)$ be the actual output state. By assumption, $\|\psi^{\otimes m}-\sigma\|_1\leq 2\epsilon_n$. Using the standard relation between the trace distance and fidelity [11], $\|\psi^{\otimes m}-\sigma\|_1\leq 2\epsilon_n$ implies that the infidelity $1-\langle\psi|^{\otimes m}\sigma|\psi\rangle^{\otimes m}\leq\epsilon_n$. Then, according to the corollary 1, there exists a pure state $|\Theta_n\rangle$ (namely the eigenvector of σ with the largest eigenvalue) which satisfies both inequalities

$$|\langle\Theta_n|\psi\rangle^{\otimes m}|^2\geq 1-2\epsilon_n\ , \tag{204}$$

and

$$P_{H_\text{tot}}(\sigma)\geq V_{H_\text{tot}}(|\Theta_n\rangle)\times(\frac{(1-\epsilon_n)^2}{\epsilon_n}-1)\ , \tag{205}$$

where $H_\text{tot}=\sum_{i=1}^m H^{(i)}$ is the sum of the Hamiltonians of the output systems.

Therefore, using the monotonicity of the purity of coherence, we conclude

$$V_{H_\text{tot}}(|\Theta_n\rangle)\times(\frac{(1-\epsilon_n)^2}{\epsilon_n}-1)\leq P_{H_\text{tot}}(\sigma)\leq nP_H(\rho)+\frac{2(d_\chi-1)}{\epsilon_n}\,V_{H_\text{help}}(\chi)\ , \tag{206}$$

or, equivalently,

$$\epsilon_nP_H(\rho)+2(d_\chi-1)\,\frac{1}{n}V_{H_\text{help}}(\chi)\geq\frac{1}{n}V_{H_\text{tot}}(|\Theta_n\rangle)\times((1-\epsilon_n)^2-\epsilon_n)\ . \tag{207}$$

To complete the proof, in the following we prove Supplementary Eq.(202): Let τ_χ be the state obtained by mixing the pure state χ and the totally mixed state I/d_χ, such that the trace distance between τ_χ and χ is ϵ. Then,

$$\tau_\chi=(1-\frac{\epsilon}{2})|\chi\rangle\langle\chi|+\frac{\epsilon}{2(d_\chi-1)}(I-|\chi\rangle\langle\chi|)\ . \tag{208}$$

Recall that for any Hamiltonian H and state ρ with spectral decomposition $\rho=\sum_j p_j|\psi_j\rangle\langle\psi_j|$, we have $P_H(\rho)=\sum_{k,l}\frac{p_k^2-p_l^2}{p_l}|\langle\psi_k|H|\psi_l\rangle|^2$. Therefore, for any j and k whose corresponding eigenvalues are equal, the corresponding term

$\frac{p_k^2-p_l^2}{p_l}|\langle\psi_k|H|\psi_l\rangle|^2$ does not contribute in the summation. Using this for state τ_χ in Supplementary Eq.(208), we find

$$P_{H_{\text{help}}}(\tau_\chi) \leq \frac{(1-\frac{\epsilon}{2})^2-(\frac{\epsilon}{2(d_\chi-1)})^2}{\frac{\epsilon}{2(d_\chi-1)}} \sum_{l:\psi_l\neq\chi} |\langle\chi|H_{\text{help}}|\psi_l\rangle|^2 \tag{209}$$

$$\leq \frac{2(d_\chi-1)}{\epsilon} V_{H_{\text{help}}}(\chi) \ . \tag{210}$$

This proves Supplementary Eq.(202) and completes the proof of the lemma.

Supplementary Note 6: Mixed states with distillable coherence

In this section we study examples of mixed states for which the distillable coherence is non-zero.

First, we consider states in the form

$$\rho = p|\psi\rangle\langle\psi| + (1-p)\sigma \ , \tag{211}$$

where $0 < p < 1$. We assume the support of σ is limited to a proper subspace of the Hilbert space, and the pure state ψ does not belong to this subspace.

Consider the subspace spanned by all the energy levels $\{|E_i\rangle : \langle E_i|\sigma|E_i\rangle > 0\}$, i.e. energy levels in which σ has a non-zero probability. Let P_σ be the projector to this subspace, and $P_\sigma^\perp = I - P_\sigma$ be the projector to the orthogonal subspace. This means that

$$[P_\sigma^\perp, H] = 0, \quad \text{and,} \quad P_\sigma^\perp \sigma = \sigma P_\sigma^\perp = 0 \ . \tag{212}$$

Then, the two-outcome projective measurement $\{P_\sigma^\perp, P_\sigma\}$ is a TI operation. By performing this TI operation on the input state ρ, we project the system to the pure state

$$|\tilde{\psi}\rangle = \frac{P_\sigma^\perp|\psi\rangle}{\sqrt{\langle\psi|P_\sigma^\perp|\psi\rangle}} \ , \tag{213}$$

with probability

$$\text{Tr}(\rho P_\sigma^\perp) = p \times \langle\psi|P_\sigma^\perp|\psi\rangle \ . \tag{214}$$

From results of [3, 12–14] we know that copies of state $|\tilde{\psi}\rangle$ can be transformed to copies of any other pure state ϕ_{coh}, which has the same period, with the optimal rate $V_H(\tilde{\psi})/V_H(\phi_{\text{coh}})$, where $V_H(\tilde{\psi}) = \langle\tilde{\psi}|H^2|\tilde{\psi}\rangle - \langle\tilde{\psi}|H|\tilde{\psi}\rangle^2$, is the energy variance.

It follows that using this strategy, we obtain copies of state ϕ_{coh} from states ρ with the rate

$$R = p \times \langle\psi|P_\sigma^\perp|\psi\rangle \times \frac{V_H(\tilde{\psi})}{V_H(\phi_{\text{coh}})} \ . \tag{215}$$

In the special case where σ is incoherent, i.e. $[\sigma, H] = 0$, it turns out that this rate can be written nicely in terms of the function

$$Q_H(\rho) \equiv \text{Tr}(H\rho H \Pi_\rho^\perp) \ , \tag{216}$$

where $\Pi_\rho^\perp = I - \Pi_\rho$ is the projector to the kernel of ρ. As we show later, $Q_H(\rho)$ is closely related to Petz-Rényi relative entropies. Also, note that for any pure state ϕ, this function is equal to energy variance, i.e. $Q_H(\phi) = V_H(\phi)$. In the following, we show that if σ is incoherent, then for state $\rho = p|\psi\rangle\langle\psi| + (1-p)\sigma$,

$$Q_H(\rho) = p \times \langle\psi|P_\sigma^\perp|\psi\rangle \times V_H(\tilde{\psi}) \ , \tag{217}$$

and therefore, the above rate R can be rewritten as

$$R = p \times \langle\psi|P_\sigma^\perp|\psi\rangle \times \frac{V_H(\tilde{\psi})}{V_H(\phi_{\text{coh}})} \tag{218}$$

$$= \frac{Q_H(\rho)}{V_H(\phi_{\text{coh}})} \tag{219}$$

$$= \frac{Q_H(\rho)}{Q_H(\phi_{\text{coh}})} \ . \tag{220}$$

To see Supplementary Eq.217, note that if σ is incoherent, then it can be diagonalized in the energy eigenbasis, as

$$\sigma = \sum_{i\in S} q_i |E_i\rangle\langle E_i| \ , \tag{221}$$

where the summation is over all energy eigenstates with non-zero probability, i.e. $q_i > 0$. Then, the support of $\rho = p|\psi\rangle\langle\psi| +$

$(1-p)\sigma$ is the subspace spanned by $\{|E_i\rangle : i \in S\} \cup \{|\psi\rangle\}$. Since,

$$P_\sigma = \sum_{i\in S} |E_i\rangle\langle E_i| \ . \tag{222}$$

and $P_\sigma^\perp = I - P_\sigma$, the subspace spanned by $\{|E_i\rangle : i \in S\} \cup \{|\psi\rangle\}$ is equal to the subspace spanned by $\{|E_i\rangle : i \in S\} \cup \{P_\sigma^\perp|\psi\rangle\}$. Therefore, the projector to the support of ρ is

$$\Pi_\rho = P_\sigma + |\tilde{\psi}\rangle\langle\tilde{\psi}| \ , \tag{223}$$

and the projector to its kernel is

$$\Pi_\rho^\perp = I - \Pi_\rho = P_\sigma^\perp - |\tilde{\psi}\rangle\langle\tilde{\psi}|. \tag{224}$$

Note that

$$\Pi_\rho^\perp = P_\sigma^\perp \Pi_\rho^\perp P_\sigma^\perp \ . \tag{225}$$

Using this formula we have

$$\begin{aligned}
Q_H(\rho) \equiv \text{Tr}(H\rho H \Pi_\rho^\perp) &= \text{Tr}\Big(H[p|\psi\rangle\langle\psi| + (1-p)\sigma]H[P_\sigma^\perp \Pi_\rho^\perp P_\sigma^\perp]\Big) & (226)\\
&= p\text{Tr}\Big(H|\psi\rangle\langle\psi|H[P_\sigma^\perp \Pi_\rho^\perp P_\sigma^\perp]\Big) + (1-p)\text{Tr}\Big(H^2\sigma[P_\sigma^\perp \Pi_\rho^\perp P_\sigma^\perp]\Big) & (227)\\
&= p\text{Tr}\Big(HP_\sigma^\perp|\psi\rangle\langle\psi|P_\sigma^\perp H \Pi_\rho^\perp\Big) & (228)\\
&= p \times \langle\psi|P_\sigma^\perp|\psi\rangle \times \text{Tr}\Big(H|\tilde{\psi}\rangle\langle\tilde{\psi}|H\Pi_\rho^\perp\Big) & (229)\\
&= p \times \langle\psi|P_\sigma^\perp|\psi\rangle \times \text{Tr}\Big(H|\tilde{\psi}\rangle\langle\tilde{\psi}|H[P_\sigma^\perp - |\tilde{\psi}\rangle\langle\tilde{\psi}|]\Big) & (230)\\
&= p \times \langle\psi|P_\sigma^\perp|\psi\rangle \times V_H(\tilde{\psi}) \ , & (231)
\end{aligned}$$

where to get the second line we have used the fact that $[H,\sigma] = 0$, to get the third line we have used the fact that $P_\sigma^\perp\sigma = \sigma P_\sigma^\perp = 0$, to get the fourth line we have used the definition $|\tilde{\psi}\rangle = P_\sigma^\perp|\psi\rangle/\sqrt{\langle\psi|P_\sigma^\perp|\psi\rangle}$, to get the fifth line we have used Supplementary Eq.224, and to get the last line we have the facts that $P_\sigma^\perp$ commutes with H, and $P_\sigma^\perp|\tilde{\psi}\rangle = |\tilde{\psi}\rangle$. This proves Supplementary Eq.217.

It is worth noting that function $Q_H(\rho)$ can be obtained from Petz-Rényi relative entropies, in the same way we derived the purity of coherence: For $\alpha = 0$, the Petz-Rényi relative entropy can be defined by taking the limit $\alpha \to 0$ of

$$D_\alpha(\rho\|\sigma) = \frac{1}{\alpha - 1}\log\text{Tr}(\rho^\alpha\sigma^{1-\alpha}) \ , \tag{232}$$

which yields

$$D_0(\rho\|\sigma) = -\log\text{Tr}(\Pi_\rho\sigma) \ . \tag{233}$$

Choosing $\sigma = e^{-iH\Delta t}\rho e^{iH\Delta t}$, for small Δt, we find

$$D_0(\rho\|\sigma) = (\Delta t)^2 \times [\text{Tr}(\rho H^2) - \text{Tr}(H\rho H\Pi_\rho)] + \mathcal{O}(\Delta t^4) = (\Delta t)^2 \times Q_H(\rho) + \mathcal{O}(\Delta t^4) \ . \tag{234}$$

In other words,

$$Q_H(\rho) = \frac{1}{2}\frac{d^2}{dt^2} D_0(\rho\|e^{-iH\Delta t}\rho e^{iH\Delta t})\Big|_{t=0} \ . \tag{235}$$

Then, following the same arguments we used in the case of the purity of coherence, one can easily show that $Q_H(\rho)$ is additive and monotone under TI operations. Furthermore, $Q_H(\rho)$ is zero, iff $[\Pi_\rho, H] = 0$, i.e. iff the purity of coherence of ρ is bounded. To see this note that $Q_H(\rho) > 0$, iff $H\rho H$ has support in the kernel of ρ. On the other hand, $P_H(\rho) = \text{Tr}(H\rho^2 H\rho^{-1}) - \text{Tr}(H^2\rho) = \infty$, iff $H\rho^2 H$ has support in the kernel of ρ. Because the support of $H\rho H$ is equal to the support of $H\rho^2 H$, then these two conditions are equivalent.

Generalization of the above example

The previous example can be generalized extensively. Specifically, suppose the Hilbert space $\mathcal{H}$ of the system with Hamiltonian H and state ρ can be decomposed to

$$\mathcal{H} = \bigoplus_{\mu} \mathcal{H}_\mu^{\text{pure}} \otimes \mathcal{H}_\mu^{\text{mixed}} \ , \tag{236}$$

such that

(i) Hamiltonian H is block-diagonal with respect to the subspaces $\{\mathcal{H}_\mu^{\text{pure}} \otimes \mathcal{H}_\mu^{\text{mixed}}\}_\mu$, i.e.

$$\forall \mu : \ [H, \Pi_\mu] = 0 \ , \tag{237}$$

where Π_μ is the projector to the subspace $\mathcal{H}_\mu^{\text{pure}} \otimes \mathcal{H}_\mu^{\text{mixed}}$.

(ii) Furthermore, the operator $\Pi_\mu H \Pi_\mu$ does not introduce interactions between subsystems $\mathcal{H}_\mu^{\text{pure}}$ and $\mathcal{H}_\mu^{\text{mixed}}$ (i.e. it can be written as the sum of two terms, each acting non-trivially on, at most, one of $\mathcal{H}_\mu^{\text{mixed}}$ and $\mathcal{H}_\mu^{\text{pure}}$).

(iii) The reduced (unnormalized) state of $\mathcal{H}_\mu^{\text{pure}}$, defined by

$$|\psi_\mu\rangle\langle\psi_\mu| = \text{Tr}_{\mathcal{H}_\mu^{\text{mixed}}}(\Pi_\mu \rho \Pi_\mu) \ , \tag{238}$$

is a pure state, and the reduced (unnormalized) state of $\mathcal{H}_\mu^{\text{mixed}}$, defined by

$$\tau_\mu = \text{Tr}_{\mathcal{H}_\mu^{\text{pure}}}(\Pi_\mu \rho \Pi_\mu) \ , \tag{239}$$

is a mixed state. Note that we can always choose $\mathcal{H}_\mu^{\text{pure}}$ or $\mathcal{H}_\mu^{\text{mixed}}$ to be one-dimensional subsystems. For one-dimensional subsystems, the unnormalized reduced state is both pure and mixed.

Suppose for a subspace corresponding to label μ^*, the reduced state of the subsystems $\mathcal{H}_{\mu^*}^{\text{pure}}$, i.e. state $|\psi_{\mu^*}\rangle\langle\psi_{\mu^*}|$, contains coherence, i.e. does not commute with the Hamiltonian induced on $\mathcal{H}_{\mu^*}^{\text{pure}}$. Then, by performing the projective measurement corresponding to projectors $\{\Pi_\mu\}_\mu$, which is a TI operation, and discarding the subsystem $\mathcal{H}_\mu^{\text{mixed}}$, we obtain the (unnormalized) pure state $|\psi_{\mu^*}\rangle\langle\psi_{\mu^*}|$. Then, because this pure state contains coherence, we can use it to distill coherence. Therefore, in this case the distillable coherence for state ρ is non-zero.

The question of classifying all states with non-zero distillable coherence remains open. In particular, it is not clear if the above family of states includes all states with non-zero distillable coherence.

Supplementary Note 7: Sub-linear distillation with a measure-and-prepare TI process (Proof of Eq. 14 in the paper)

In this section we study a coherence distillation process which works based on a measure-and-prepare TI process.

Covariant estimators

Consider the following parameter estimation problem: Suppose we are given n copies of state $e^{-iHt}\rho e^{iHt}$, where $t \in [0, \tau)$ is unknown, and τ is the period. By performing a measurement on these systems we can find an estimate $t_{\text{est}} \in [0, \tau)$ of t, with probability density $p(t_{\text{est}}|t)$. This estimator can be described by the POVM

$$\{M_{t_{\text{est}}} dt_{\text{est}} : \ t_{\text{est}} \in [0, \tau)\} ,$$

such that

$$p(t_{\text{est}}|t) = \text{Tr}(M_{t_{\text{est}}}(e^{-iHt}\rho e^{iHt})^{\otimes n}) . \tag{240}$$

Given any such POVM, we can construct a new POVM defined by

$$\tilde{M}_{t_{\text{est}}} = \frac{1}{\tau}\int_0^{\tau} ds\ (e^{iHs})^{\otimes n} M_{t_{\text{est}}+s}(e^{-iHs})^{\otimes n} , \tag{241}$$

where $t_{\text{est}} + s$ is mod τ. This POVM describes the estimator which first shifts the received state state by $(e^{-iHs})^{\otimes n}$, where s is chosen uniformly at random, then apply the original estimator, and at the end, cancels the shift s at the output.

Such estimators are guaranteed to be invariant under time-translations in the following sense

$$(e^{iHr})^{\otimes n}\tilde{M}_{t_{\text{est}}+r}(e^{-iHr})^{\otimes n} = \tilde{M}_{t_{\text{est}}} \ : \forall r \in [0, \tau) , \tag{242}$$

where $t_{\text{est}} + r$ is mod τ. The probability density of outcome t_{est} for this POVM, i.e.

$$\tilde{p}(t_{\text{est}}|t) \equiv \text{Tr}(\tilde{M}_{t_{\text{est}}}(e^{-iHt}\rho e^{iHt})^{\otimes n}) , \tag{243}$$

satisfies

$$\tilde{p}(t_{\text{est}}|t) = \frac{1}{\tau}\int_0^{\tau} ds\, p(t_{\text{est}} + s|t + s) \tag{244}$$

and is invariant under time translations, i.e.

$$\tilde{p}(t_{\text{est}}|t) = \tilde{p}(t_{\text{est}} + r|t + r) \ : \forall r \in [0, \tau) . \tag{245}$$

Therefore, the Mean Squared Error (MSE) of the new estimator is independent of t, and is equal to

$$\langle \delta t^2 \rangle = \int_0^{\tau} dt_{\text{est}}\ \tilde{p}(t_{\text{est}}|t)(t - t_{\text{est}})^2 = \int_0^{\tau} dt_{\text{est}}\ \frac{1}{\tau}\int_0^{\tau} ds\, p(t_{\text{est}} + s|t + s)(t - t_{\text{est}})^2 \tag{246}$$

$$= \frac{1}{\tau}\int_0^{\tau} ds \int_0^{\tau} dr\ \ p(r|t + s)(t - r + s)^2 \tag{247}$$

$$= \frac{1}{\tau}\int_0^{\tau} ds\, \Big[\int_0^{\tau} dr\, p(r|s)(s - r)^2\Big] , \tag{248}$$

which is the average of MSE for the original estimator.

In the following we always assume that the estimator satisfies the covariance condition in Supplementary Eq.242, or equivalently, 245; otherwise, if an estimator does not satisfy this condition we can always construct a new estimator which satisfies this condition. Then, the MSE for the new estimator is independent of the parameter t, and is equal to the average MSE for the original estimator.

A TI measure-and-prepare channel

Next, suppose after estimating t we prepare m copies of state $e^{-iHt_{\rm est}}|\phi\rangle$. Overall this process implements the state transformation

$$(e^{-iHt}\rho e^{iHt})^{\otimes n} \longrightarrow \int_0^\tau dt_{\rm est}\ p(t_{\rm est}|t)\ \Big(e^{-iHt_{\rm est}}|\phi\rangle\langle\phi|e^{iHt_{\rm est}}\Big)^{\otimes m}\ , \tag{249}$$

and is described by the TI quantum channel $\mathcal{E}_{\rm TI}$, defined by

$$\mathcal{E}_{\rm TI}(\sigma) = \int_0^\tau dt_{\rm est}\ {\rm Tr}(\sigma M_{t_{\rm est}})\Big(e^{-iHt_{\rm est}}|\phi\rangle\langle\phi|e^{iHt_{\rm est}}\Big)^{\otimes m}\ . \tag{250}$$

The fact that this channel is TI follows immediately from the covariance condition for POVM in Supplementary Eq.242.

Applying this TI channel to input $\rho^{\otimes n}$, we obtain

$$\mathcal{E}_{\rm TI}(\rho^{\otimes n}) = \int_0^\tau dt_{\rm est}\ {\rm Tr}(\rho^{\otimes n} M_{t_{\rm est}})\Big(e^{-iHt_{\rm est}}|\phi\rangle\langle\phi|e^{iHt_{\rm est}}\Big)^{\otimes m} = \int_0^\tau dt_{\rm est}\ p(t_{\rm est}|t=0)\ \Big(e^{-iHt_{\rm est}}|\phi\rangle\langle\phi|e^{iHt_{\rm est}}\Big)^{\otimes m}\ , \tag{251}$$

where

$$p(t_{\rm est}|t=0) = {\rm Tr}(M_{t_{\rm est}}\rho^{\otimes n})\ . \tag{252}$$

The fidelity of this state with $|\phi\rangle^{\otimes m}$ is

$$\langle\phi|^{\otimes m}\mathcal{E}_{\rm TI}(\rho^{\otimes n})|\phi\rangle^{\otimes m} = \int_0^\tau dt_{\rm est}\ p(t_{\rm est}|t=0)\ |\langle\phi|e^{iHt_{\rm est}}|\phi\rangle|^{2m}\ . \tag{253}$$

To bound this fidelity, we use the fact that

$$|\langle\phi|e^{iHr}|\phi\rangle|^{2m} \geq 1 - r^2 m \times V_H(\phi)\ , \tag{254}$$

which is proven at the end of this section. Using this bound, we find

$$\langle\phi|^{\otimes m}\mathcal{E}_{\rm TI}(\rho^{\otimes n})|\phi\rangle^{\otimes m} = \int_0^\tau dt_{\rm est}\ p(t_{\rm est}|t=0)\ |\langle\phi|e^{iHt_{\rm est}}|\phi\rangle|^{2m} \tag{255}$$

$$\geq \int_0^\tau dt_{\rm est}\ p(t_{\rm est}|t=0) \times [1 - t_{\rm est}^2 m \times V_H(\phi)] \tag{256}$$

$$= 1 - m \times V_H(\phi) \times \langle\delta t^2\rangle \tag{257}$$

where $\langle\delta t^2\rangle = \int_0^\tau dt_{\rm est}\ p(t_{\rm est}|t)(t - t_{\rm est})^2$ is the MSE of the estimator and is independent of t (Recall that we have assumed the estimator is invariant under time translations).

Let

$$\epsilon_n = 1 - \langle\phi|^{\otimes m}\mathcal{E}_{\rm TI}(\rho^{\otimes n})|\phi\rangle^{\otimes m}\ , \tag{258}$$

be the infidelity between state $|\phi\rangle^{\otimes m}$ and $\mathcal{E}_{\rm TI}(\rho^{\otimes n})$. Then, the above result means that

$$\frac{m}{\epsilon_n} \geq \frac{1}{V_H(\phi) \times \langle\delta t^2\rangle}\ . \tag{259}$$

Diving both sides by n, we find

$$\frac{r(n)}{\epsilon_n} \geq \frac{1}{V_H(\phi) \times n \times \langle\delta t^2\rangle}\ , \tag{260}$$

where $r(n) = m(n)/n$ is the yield. Finally, using $F_H(\phi) = 4 \times V_H(\phi)$, we find

$$\frac{r(n)}{\epsilon_n} \geq \frac{4}{F_H(\phi) \times n \times \langle\delta t^2\rangle}\ . \tag{261}$$

The MSE $\langle \delta t^2 \rangle$ for any reasonable estimator scales as $1/n$. Therefore, as n goes to infinity, the above lower bound remains a positive non-zero constant. In particular, as shown in [15, 16], there exists an estimator, based on the Maximum Likelihood (ML) estimator, which achieves MSE equal to

$$\langle \delta t^2 \rangle = \frac{1}{nF_H(\rho)} + o(\frac{1}{n}) , \tag{262}$$

i.e. saturates the Quantum Cramér-Rao bound [15, 17, 18]. Using this estimator we obtain

$$\frac{r(n)}{\epsilon_n} \geq 4\frac{F_H(\rho)}{F_H(\phi)} \times [1 - o(1)] , \tag{263}$$

which is the lower bound on maximum achievable yield in Eq. 13 of the main paper. To complete the proof, we need to prove the bound in Supplementary Eq.254, which is presented in the following section.

It is worth noting that if the Hamiltonian H is bounded and m is fixed, then the above bound is tight, up to corrections of $o(1/n)$. In other words, the measure-and-prepare TI process working based on the ML estimator transforms input $\rho^{\otimes n}$ to an output state whose infidelity with $|\phi\rangle^{\otimes m}$ is equal to

$$\epsilon_n = mV_H(\phi) \times \langle \delta t^2 \rangle + o(\frac{1}{n}) = \frac{1}{n} \times \frac{mF_H(\phi)}{4F_H(\rho)} + o(\frac{1}{n}) . \tag{264}$$

To see this note that for ML estimator (as well as any other reasonable estimator) the second movement $\langle \delta t^2 \rangle$ scales as $1/n$, and the higher moments scales as $o(1/n)$. Hence, assuming the Hamiltonian H is bounded and m is fixed, the Supplementary Eq.257 holds as equality, up to correction of order $o(1/n)$, i.e. $\epsilon_n = mV_H(\phi) \times \langle \delta t^2 \rangle + o(\frac{1}{n})$ (This can be seen by Taylor expanding $|\langle \phi | e^{iHt_{\text{est}}} | \phi \rangle|^{2m}$ in powers of t_{est}). The second equality in Eq.(264) follows from the fact that ML estimator asymptotically achieves the Quantum Cramér-Rao bound, up to corrections of order $o(1/n)$.

Proof of Supplementary Eq.254

In the following, we show that for any Hamiltonian G, state $|\eta\rangle$, and $r \in \mathbb{R}$,

$$|\langle \eta | e^{iGr} | \eta \rangle|^2 \geq 1 - r^2 V_G(\eta) , \tag{265}$$

where $V_G(\eta) = \langle \eta | G^2 | \eta \rangle - \langle \eta | G | \eta \rangle^2$. To see this note that

$$\left| \langle \eta | e^{iGr} | \eta \rangle \right|^2 = 1 + \int_0^r ds_1 \int_0^{s_1} ds_2 \, \frac{d^2}{ds_2^2} \left| \langle \eta | e^{iGs_2} | \eta \rangle \right|^2 \tag{266}$$

$$\geq 1 + \frac{r^2}{2} \times \min_{s_2 \in [0,s]} \frac{d^2}{ds_2^2} \left| \langle \eta | e^{iGs_2} | \eta \rangle \right|^2 . \tag{267}$$

Then, we note that

$$\frac{d}{ds} |\langle \eta | e^{iGs} | \eta \rangle|^2 = \frac{d}{ds} \text{Tr}(|\eta\rangle\langle\eta| e^{-iGs} |\eta\rangle\langle\eta| e^{iGs}) \tag{268}$$

$$= -i \text{Tr}(|\eta\rangle\langle\eta| \Big[G, e^{-iGs} |\eta\rangle\langle\eta| e^{iGs} \Big]) \tag{269}$$

$$= -i \text{Tr}(\Big[|\eta\rangle\langle\eta|, G \Big] e^{-iGs} |\eta\rangle\langle\eta| e^{iGs}) . \tag{270}$$

Therefore,

$$\frac{d^2}{ds^2} |\langle \eta | e^{iGs} | \eta \rangle|^2 = -i \frac{d}{ds} \text{Tr}(\Big[|\eta\rangle\langle\eta|, G \Big] e^{-iGs} |\eta\rangle\langle\eta| e^{iGs}) \tag{271}$$

$$= \text{Tr}(\Big[|\eta\rangle\langle\eta|, G \Big] e^{-iGs} \Big[|\eta\rangle\langle\eta|, G \Big] e^{iGs}) \tag{272}$$

Therefore,

$$\left|\frac{d^2}{ds^2}|\langle\eta|e^{iGs}|\eta\rangle|^2\right| = \left|\mathrm{Tr}(\Big[|\eta\rangle\langle\eta|, G\Big]e^{-iGs}\Big[|\eta\rangle\langle\eta|, G\Big]e^{iGs})\right| \tag{273}$$

$$\leq \left|\mathrm{Tr}(\Big[|\eta\rangle\langle\eta|, G\Big]^2)\right| \tag{274}$$

$$= 2V_G(\eta)\ , \tag{275}$$

where the bound follows from Cauchy-Schwartz inequality. This means that

$$\min_{s_2\in[0,s]} \frac{d^2}{ds_2^2}\left|\langle\eta|e^{iGs_2}|\eta\rangle\right| \geq -2V_G(\eta)\ . \tag{276}$$

Putting this back into Supplementary Eq.267 we find Supplementary Eq.265 .

Now consider m copies of a system with state $|\phi\rangle$ and Hamiltonian H, i.e. a composite system with the joint state $|\eta\rangle = |\phi\rangle^{\otimes m}$, and the total Hamiltonian $H_{\text{tot}} = \sum_{i=1}^{m} H^{(i)}$. Then, the total energy variance with respect to the total Hamiltonian H_{tot} is $m \times V_H(\phi)$. Therefore, we conclude that

$$|\langle\phi|^{\otimes m}e^{iH_{\text{tot}}r}|\phi\rangle^{\otimes m}|^2 = |\langle\phi|e^{iHr}|\phi\rangle|^{2m} \geq 1 - r^2 m \times V_H(\phi)\ , \tag{277}$$

which proves Supplementary Eq.254.

Supplementary Note 8: Purity of coherence of the output of Measure-and-Prepare TI channels is upper bounded by QFI of the input

Recall that a quantum channel is called a Measure-and-Prepare channel if it can be written as

$$\mathcal{E}_{\text{MP}}(\rho) = \sum_{x\in\mathcal{X}} \text{Tr}(M_x\rho)\sigma_x \ , \tag{278}$$

where $\{M_x : x \in \mathcal{X}\}$ is a POVM and $\{\sigma_x : x \in \mathcal{X}\}$ is a set of density operators [11]. Furthermore, assuming the input Hilbert space is finite-dimensional, the set of outcomes $\mathcal{X}$ can be chosen to have finite elements [11]. It turns out that a quantum channel is Measure-and-Prepare if, and only if, it is entanglement-breaking.

In this section we prove that for any TI Measure-and-Prepare process $\mathcal{E}_{\text{TI-MP}}$, and any input ρ it holds that

$$P_{H_{\text{out}}}(\mathcal{E}_{\text{TI-MP}}(\rho)) \leq F_{H_{\text{in}}}(\rho) \leq P_{H_{\text{in}}}(\rho) \ , \tag{279}$$

where H_{in} and H_{out} are, respectively, the input and output Hamiltonians. We have shown the inequality $F_{H_{\text{in}}}(\rho) \leq P_{H_{\text{in}}}(\rho)$ before. In the following we prove $P_{H_{\text{out}}}(\mathcal{E}_{\text{TI-MP}}(\rho)) \leq F_{H_{\text{in}}}(\rho)$.

Define the channels

$$\mathcal{E}_{\text{meas}}(\cdot) = \sum_{x\in\mathcal{X}} \text{Tr}(M_x\cdot)|x\rangle\langle x| \tag{280}$$

and

$$\mathcal{E}_{\text{prep}}(\cdot) = \sum_{x\in\mathcal{X}} \text{Tr}(|x\rangle\langle x|\cdot)\sigma_x \ , \tag{281}$$

where $\{|x\rangle\}_x$ is a set of orthonormal states. Then, for any input state ρ, we have

$$\sigma = \mathcal{E}_{\text{MP}}(\rho) = \mathcal{E}_{\text{prep}} \circ \mathcal{E}_{\text{meas}}(\rho) = \sum_{x\in\mathcal{X}} \text{Tr}(M_x\rho)\sigma_x \ . \tag{282}$$

For arbitrary time t, define

$$\rho(t) \equiv e^{-iH_{\text{in}}t}\rho e^{iH_{\text{in}}t} \ , \tag{283}$$

$$\omega_{\text{cl}}(t) \equiv \mathcal{E}_{\text{meas}}(\rho(t)) = \sum_{x\in\mathcal{X}} p_t(x)|x\rangle\langle x| \ , \tag{284}$$

$$\sigma(t) \equiv e^{-iH_{\text{out}}t}\sigma e^{iH_{\text{out}}t} = \mathcal{E}_{\text{MP-TI}}(\rho(t)) = \mathcal{E}_{\text{prep}}(\omega_{\text{cl}}(t)) \ , \tag{285}$$

where

$$p_t(x) = \text{Tr}(M_x\rho(t)) \ . \tag{286}$$

To summarize, we have

$$\forall t : \quad \rho(t) \xrightarrow{\text{Measurement}} \omega_{\text{cl}}(t) \xrightarrow{\text{Preperation}} \sigma(t) \ . \tag{287}$$

In the following, we show that

$$F_{H_{\text{in}}}(\rho) \geq I_{t=0} \geq P_{H_{\text{out}}}(\sigma) \ , \tag{288}$$

where $I_{t=0}$ is the classical Fisher information for the family of distribution p_t, corresponding to parameter t, at $t = 0$. We note that the bound $I_{t=0} \geq P_{H_{\text{out}}}(\sigma)$ also follows from the general result of [19]. The bound $F_{H_{\text{in}}}(\rho) \geq I_{\text{t=0}}$ is a consequence of the monotonicity of QFI under data processing. Here, for completeness, we prove this directly using the relation between QFI and the fidelity.

Before presenting the proof, we note that if the input Hilbert space is finite-dimensional and the Hamiltonian is bounded, then the output probabilities $p_t(x)$ are analytic functions of t. Furthermore, without loss of generality we can assume for any outcome $x \in \mathcal{X}$, outcome probability $p_t(x)$ is non-zero for some t; otherwise, we can combine all the POVM elements with zero probability with a POVM element with non-zero probability, without changing the action of the channel $\mathcal{E}_{\text{TI-MP}}$ on the input state ρ. Moreover, we assume for all outcomes $x \in \mathcal{X}$, the corresponding probabilities $p_t(x)$ are non-zero at $t = 0$;

if this is not the case, then we can shift the point $t = 0$ by a properly chosen $s \in \mathbb{R}$, or equivalently, we can replace the input ρ with its translated version, namely state $\rho(s) = e^{-iH_{\text{in}}s}\rho e^{iH_{\text{in}}s}$, where $s \in \mathbb{R}$ is chosen such that the probabilities $p_s(x) = \text{Tr}(M_x\rho(s)) > 0$ for all outcomes $x \in \mathcal{X}$ (Since all the probabilities $\{p_s(x) : x \in \mathcal{X}\}$ are non-zero analytic functions of s, and $\mathcal{X}$ is a finite set, there always exists $s \in \mathbb{R}$, which satisfies this property). Then, the following argument proves that $P_{H_{\text{out}}}(\mathcal{E}_{\text{TI-MP}}(e^{-iH_{\text{out}}s}\rho e^{iH_{\text{out}}s})) \le F_{H_{\text{in}}}(e^{-iH_{\text{in}}s}\rho e^{iH_{\text{in}}s})$, which immediately implies $P_{H_{\text{out}}}(\mathcal{E}_{\text{TI-MP}}(\rho)) \le F_{H_{\text{in}}}(\rho)$. Therefore, without loss of generality, in the following we assume

$$p(x) \equiv p_{t=0}(x) \equiv \text{Tr}(M_x\rho) > 0, \ \forall x \in \mathcal{X} \ . \tag{289}$$

Recall that the fidelity between two states ρ_1 and ρ_2 is defined as[25], $\text{Fid}(\rho_1, \rho_2) = \|\sqrt{\rho_1}\sqrt{\rho_2}\|_1^2$. Then, QFI is equal to

$$F_{H_{\text{in}}}(\rho) = -4\frac{d^2}{dt^2}\sqrt{\text{Fid}(\rho, \rho(t))}\ \Big|_{t=0} \ . \tag{290}$$

Note that the first derivative vanishes at $t = 0$.

Recall that fidelity is monotone under CPTP maps, i.e. for any quantum channel $\mathcal{E}$,

$$\text{Fid}(\mathcal{E}(\rho_1), \mathcal{E}(\rho_2)) \ge \text{Fid}(\rho_1, \rho_2) \ . \tag{291}$$

It follows that for any quantum channel $\mathcal{E}$,

$$F_{H_{\text{in}}}(\rho) = -4\frac{d^2}{dt^2}\sqrt{\text{Fid}(\rho, \rho(t))}\ \Big|_{t=0} \ge -4\frac{d^2}{dt^2}\sqrt{\text{Fid}(\mathcal{E}(\rho), \mathcal{E}(\rho(t)))}\ \Big|_{t=0} \ . \tag{292}$$

We apply this to channel $\mathcal{E}_{\text{meas}}$. For this channel we have,

$$\sqrt{\text{Fid}(\mathcal{E}_{\text{meas}}(\rho), \mathcal{E}_{\text{meas}}(\rho(t)))} = \sum_{x\in\mathcal{X}} \sqrt{p(x)p_t(x)} \ . \tag{293}$$

Let $\dot{p}_t(x) = \frac{d}{dt}p_t(x)$ and $\ddot{p}_t(x) = \frac{d^2}{dt^2}p_t(x)$ be, respectively, the first and second derivatives of $p_t(x)$ with respect to time t. Then,

$$\frac{d^2}{dt^2}\sqrt{\text{Fid}(\mathcal{E}_{\text{meas}}(\rho), \mathcal{E}_{\text{meas}}(e^{-iHt}\rho e^{iHt}))} = \frac{d^2}{dt^2}\sum_{x\in\mathcal{X}} \sqrt{p(x)p_t(x)} \tag{294}$$

$$= \frac{d}{dt}\Big(\frac{d}{dt}\sum_{x\in\mathcal{X}} \sqrt{p(x)p_t(x)}\Big) \tag{295}$$

$$= \frac{1}{2}\frac{d}{dt}\Big(\sum_{x\in\mathcal{X}} \sqrt{p(x)}\dot{p}_t(x)[p_t(x)]^{-1/2}\Big) \tag{296}$$

$$= -\frac{1}{4}\sum_{x\in\mathcal{X}} \sqrt{p(x)}\dot{p}_t^2(x)[p_t(x)]^{-3/2} + \frac{1}{2}\sum_{x\in\mathcal{X}} \sqrt{p(x)}\ddot{p}_t(x)[p_t(x)]^{-1/2} \ . \tag{297}$$

At $t = 0$, we find

$$\frac{d^2}{dt^2}\sqrt{\text{Fid}(\mathcal{E}_{\text{meas}}(\rho), \mathcal{E}_{\text{meas}}(e^{-iHt}\rho e^{iHt}))}\ \Big|_{t=0} = -\frac{1}{4}\sum_{x\in\mathcal{X}} \sqrt{p(x)}\dot{p}_t^2(x)[p_t(x)]^{-3/2}\Big|_{t=0} + \frac{1}{2}\sum_{x\in\mathcal{X}} \sqrt{p(x)}\ddot{p}_t(x)[p_t(x)]^{-1/2}\Big|_{t=0} \ . \tag{298}$$

$$= -\frac{1}{4}\sum_{x\in\mathcal{X}} \frac{\dot{p}_t^2(x)}{p(x)}\Big|_{t=0} + \frac{1}{2}\sum_{x\in\mathcal{X}} \ddot{p}_t(x)\Big|_{t=0} \tag{299}$$

$$= -\frac{1}{4}\sum_{x\in\mathcal{X}} \frac{\dot{p}_t^2(x)}{p(x)}\Big|_{t=0} \ , \tag{300}$$

where in the last step we have used the fact that $\sum_{x\in\mathcal{X}} p_t(x) = 1$ for all t, and therefore $\sum_{x\in\mathcal{X}} \ddot{p}_t(x) = 0$. We conclude that

$$F_{H_{\text{in}}}(\rho) = -4\frac{d^2}{dt^2}\sqrt{\text{Fid}(\rho, e^{-iHt}\rho e^{iHt})}\ \Big|_{t=0} \tag{301}$$

$$\geq -4\frac{d^2}{dt^2}\sqrt{\text{Fid}(\mathcal{E}_{\text{meas}}(\rho), \mathcal{E}_{\text{meas}}(e^{-iHt}\rho e^{iHt}))}\ \Big|_{t=0} \tag{302}$$

$$= \sum_{x\in\mathcal{X}} \frac{\dot{p}_t^2(x)}{p(x)}\Big|_{t=0} . \tag{303}$$

The quantity in the last line is in fact $I_{t=0}$, the (classical) Fisher information associated to parameter t, for the family of probability distributions p_t, at $t = 0$. The above calculation basically shows that the classical Fisher information at the output of $\mathcal{E}_{\text{meas}}$ is upper bound by the Quantum Fisher information at the input. To summarize, we explicitly checked that

$$F_{H_{\text{in}}}(\rho) \geq I_{t=0} \equiv \sum_{x\in\mathcal{X}} \frac{\dot{p}_t^2(x)}{p(x)}\Big|_{t=0} . \tag{304}$$

Next, we prove the bound $I_{t=0} \geq P_{H_{\text{out}}}(\sigma)$ (See also [19]). First, recall the connection between the purity of coherence and Petz-Rényi relative entropy. In particular, using Supplementary Eq.56, the purity of coherence of $\sigma = \mathcal{E}_{\text{MP-TI}}(\rho)$ is given by

$$P_{H_{\text{out}}}(\sigma) = \frac{1}{2}\frac{d^2}{dt^2}\left[\overline{Q}\Big(\sigma\|\sigma(t)\Big)\right]_{t=0} = \frac{1}{2}\frac{d^2}{dt^2}\left[\overline{Q}\Big(\sigma(t)\|\sigma\Big)\right]_{t=0} , \tag{305}$$

where

$$\overline{Q}_2(\rho_1\|\rho_2) = \text{Tr}(\rho_1^2\rho_2^{-1}) , \tag{306}$$

is monotone under CPTP maps, i.e. for any CPTP map $\mathcal{E}$, we have

$$\overline{Q}_2(\mathcal{E}(\rho_1)\|\mathcal{E}(\rho_2)) \leq \overline{Q}_2(\rho_1\|\rho_2) . \tag{307}$$

Recall that $\sigma(t) = \mathcal{E}_{\text{prep}}(\omega_{\text{cl}}(t))$. Applying Supplementary Eq.307 to channel $\mathcal{E}_{\text{prep}}$, and the input states $\omega_{\text{cl}}(t)$ and $\omega_{\text{cl}}(0)$, we find

$$\overline{Q}_2\Big(\sigma(t)\|\sigma\Big) = \overline{Q}_2\Big(\sigma(t)\|\sigma(0)\Big) = \overline{Q}_2\Big(\mathcal{E}_{\text{prep}}(\omega_{\text{cl}}(t))\|\mathcal{E}_{\text{prep}}(\omega_{\text{cl}}(0))\Big) \leq \overline{Q}_2(\omega_{\text{cl}}(t)\|\omega_{\text{cl}}(0)) , \tag{308}$$

Taking the second derivative of both sides, and using the fact that the first derivatives vanish, we find

$$P_{H_{\text{out}}}(\sigma) \leq \frac{1}{2}\frac{d^2}{dt^2}\overline{Q}_2(\omega_{\text{cl}}(t)\|\omega_{\text{cl}}(0))\Big|_{t=0} . \tag{309}$$

Next, we note that for state $\omega_{\text{cl}}(t)$,

$$\overline{Q}_2(\omega_{\text{cl}}(t)\|\omega_{\text{cl}}(0)) = \sum_{x\in\mathcal{X}} \frac{p_t^2(x)}{p(x)} . \tag{310}$$

Taking the derivative of both sides with respect to t, we find

$$\frac{d^2}{dt^2}\overline{Q}_2(\omega_{\text{cl}}(t)\|\omega_{\text{cl}}(0))\Big|_{t=0} = \frac{d^2}{dt^2}\sum_{x\in\mathcal{X}} \frac{p_t^2(x)}{p(x)}\Big|_{t=0} \tag{311}$$

$$= 2\sum_{x\in\mathcal{X}} \frac{\dot{p}_t^2(x) + p_t(x)\ddot{p}_t(x)}{p(x)})\Big|_{t=0} \tag{312}$$

$$= 2\sum_{x\in\mathcal{X}} \frac{\dot{p}_t^2(x)}{p(x)}\Big|_{t=0} + 2\sum_{x\in\mathcal{X}} \ddot{p}_t(x)\Big|_{t=0} \tag{313}$$

$$= 2I_{t=0} , \tag{314}$$

where to get the last line we again used the fact that the fact that $\sum_{x\in\mathcal{X}} \ddot{p}(x) = 1$. Therefore, we conclude that

$$P_{H_{\text{out}}}(\sigma) \leq \frac{1}{2}\frac{d^2}{dt^2}\overline{Q}_2(\omega_{\text{cl}}(t)\|\omega_{\text{cl}}(0))\Big|_{t=0} = \sum_{x\in\mathcal{X}} \frac{\dot{p}_t^2(x)}{p(x)}\Big|_{t=0} = I_{t=0} \ . \tag{315}$$

Combining this with Supplementary Eq.304, we find

$$P_{H_{\text{out}}}(\sigma) \leq \sum_{x\in\mathcal{X}} \frac{\dot{p}_t^2(x)}{p(x)}\Big|_{t=0} = I_{t=0} \leq F_{H_{\text{in}}}(\rho) \ , \tag{316}$$

which completes the proof.

Supplementary Note 9: Distillation in the single-shot regime

Maximum achievable fidelity with a pure state (Proof of Eq.16 in the paper)

In this section, we use the approach of [20], to find a simple formula for the maximum achievable fidelity $\max_{\mathcal{E}_{\text{TI}}} \langle\psi|\mathcal{E}_{\text{TI}}(\rho^{\otimes n})|\psi\rangle$, in terms of the *conditional min-entropy*.

Recall the definition of the conditional min-entropy [6, 21, 22], $H_{\min}(\text{B}|\text{A})_\Omega$, of a bipartite state Ω_{AB},

$$2^{-H_{\min}(\text{B}|\text{A})_\Omega} = \inf_{\tau^{\text{A}}\geq 0}\{\text{Tr}(\tau^{\text{A}}) : \tau^{\text{A}} \otimes I^{\text{B}} \geq \Omega_{\text{AB}}\} . \tag{317}$$

Theorem 3 Let H_{A} and H_{B} be, respectively, the Hamiltonians of the input and output systems A and B. Let σ_{A} and be an arbitrary state of A and $|\psi\rangle_{\text{B}}$ be a pure state of system B. Then,

$$\max_{\mathcal{E}_{\text{TI}}} \langle\psi|\mathcal{E}_{\text{TI}}(\sigma_{\text{A}})|\psi\rangle_{\text{B}} = 2^{-H_{\min}(\text{B}|\text{A})_\Omega} = 2^{-H_{\min}(\text{B}|\text{A})_\Gamma}, \tag{318}$$

where the maximization is over the set of all TI operations, and state Ω_{AB} and Γ_{AB} are defined as

$$\Omega_{\text{AB}} = \lim_{T\to\infty}\frac{1}{T}\int_0^T dt\; e^{-iH_{\text{A}}t}\otimes e^{iH_{\text{B}}t}[\sigma_{\text{A}}\otimes|\psi\rangle\langle\psi|_{\text{B}}]e^{iH_{\text{A}}t}\otimes e^{-iH_{\text{B}}t} = \sum_E \Pi_E[\sigma_{\text{A}}\otimes|\psi\rangle\langle\psi|_{\text{B}}]\Pi_E , \tag{319}$$

$$\Gamma_{\text{AB}} = \lim_{T\to\infty}\frac{1}{T}\int_0^T dt\; e^{-iH_{\text{A}}t}\otimes e^{iH_{\text{B}}t}[\sigma_{\text{A}}\otimes|\overline{\psi}\rangle\langle\overline{\psi}|_{\text{B}}]e^{iH_{\text{A}}t}\otimes e^{-iH_{\text{B}}t} = \sum_E \Pi_E[\sigma_{\text{A}}\otimes|\overline{\psi}\rangle\langle\overline{\psi}|_{\text{B}}]\Pi_E , \tag{320}$$

where Π_E is the projector to the eigen-subspace of $H_{\text{A}}\otimes I_{\text{B}} - I_{\text{A}}\otimes H_{\text{B}}$ with energy E, and $|\overline{\psi}\rangle = \sum_i \overline{\langle E_i|\psi\rangle}|E_i\rangle = \sum_i\langle\psi|E_i\rangle|E_i\rangle$ is the complex conjugate of $|\psi\rangle$ in the eigenbasis of Hamiltonian H_{B}, denoted by $\{|E_i\rangle : i = 1,\cdots,d_{\text{B}}\}$, and d_{B} is the dimension of Hilbert space of B.

In other words, state Ω_{AB} is the state obtained by dephasing $\sigma_{\text{A}}\otimes|\psi\rangle\langle\psi|_{\text{B}}$ in the eingenbasis of the Hamiltonian $H_{\text{A}}\otimes I_{\text{B}} - I_{\text{A}}\otimes H_{\text{B}}$. Note that if the input system A is n copies of a system with Hamiltonian H and state ρ, then state Ω_{AB} will be given by

$$\sum_E \Pi_E[(\rho^{\otimes n})_{\text{A}}\otimes|\psi\rangle\langle\psi|_{\text{B}}]\Pi_E , \tag{321}$$

where Π_E is the projector to the eigen-subpaces of Hamiltonian $H_{\text{tot}}\otimes I_{\text{B}} - I_{\text{tot}}\otimes H_{\text{B}}$. Here, $H_{\text{tot}} = \sum_i H^{(i)}$ is the total Hamiltonian of the input systems, $H^{(i)} = I^{\otimes(i-1)}\otimes H\otimes I^{\otimes(n-i-1)}$, and $I_{\text{tot}} = I^{\otimes n}$ is the identity operator on the input systems.

Proof. Let B' be an auxiliary system with dimension equal to d_{B}, the dimension B.Define

$$|\gamma_{\text{BB}'}\rangle = \frac{1}{\sqrt{d_{\text{B}}}}\sum_{i=1}^{d_{\text{B}}}|E_iE_i\rangle_{\text{BB}'} \tag{322}$$

be a maximally entangled state of B and the auxiliary system B'. Then, for any pair of operators X and Y defined on B, we have $\text{Tr}(XY) = d_{\text{B}}\times\langle\gamma_{\text{BB}'}|[X\otimes Y^T]|\gamma_{\text{BB}'}\rangle$, where T denotes transpose in the energy eigenbasis, $\{|E_i\rangle_{\text{B}} : i = 1,\cdots,d_{\text{B}}\}$.

This implies that for any quantum channel $\mathcal{E}_{\text{TI}}$ we have

$$\langle\psi|\mathcal{E}_{\text{TI}}(\sigma)|\psi\rangle = d_{\text{B}}\times\langle\gamma_{\text{BB}'}|\left[\mathcal{E}_{\text{TI}}(\sigma)\otimes|\overline{\psi}\rangle\langle\overline{\psi}|\right]|\gamma_{\text{BB}'}\rangle , \tag{323}$$

where $|\overline{\psi}\rangle$ is the complex conjugate of $|\psi\rangle$ in the energy eigenbasis.

Next, we note that

$$\langle\psi|\mathcal{E}_{\rm TI}(\sigma)|\psi\rangle = d_{\rm B} \times \langle\gamma_{\rm BB'}| \left[\mathcal{E}_{\rm TI}(\sigma)\otimes|\overline{\psi}\rangle\langle\overline{\psi}|\right]|\gamma_{\rm BB'}\rangle \tag{324}$$

$$= d_{\rm B} \times \langle\gamma_{\rm BB'}|(e^{iH_{\rm B}t}\otimes e^{-iH_{\rm B}t})\left[\mathcal{E}_{\rm TI}(\sigma)\otimes|\overline{\psi}\rangle\langle\overline{\psi}|\right](e^{-iH_{\rm B}t}\otimes e^{iH_{\rm B}t})|\gamma_{\rm BB'}\rangle \tag{325}$$

$$= d_{\rm B} \times \langle\gamma_{\rm BB'}|(\mathcal{E}_{\rm TI}\otimes\mathcal{I}_{B'})\left([e^{iH_{\rm A}t}\otimes e^{-iH_{\rm B}t}][\sigma\otimes|\overline{\psi}\rangle\langle\overline{\psi}|][e^{-iH_{\rm A}t}\otimes e^{iH_{\rm B}t}]\right)|\gamma_{\rm BB'}\rangle\ , \tag{326}$$

where $\mathcal{I}_{B'}$ is the identity super-operator on B'. Here, to get the second line we have used the fact that

$$(e^{-iH_{\rm B}t}\otimes e^{iH_{\rm B}t})|\gamma_{\rm BB'}\rangle = |\gamma_{\rm BB'}\rangle\ , \tag{327}$$

and to get the last line we have used the fact that $\mathcal{E}_{\rm TI}$ satisfies the covariance condition

$$\mathcal{E}_{\rm TI}(e^{-iH_{\rm A}t}(\cdot)e^{iH_{\rm A}t}) = e^{-iH_{\rm B}t}\mathcal{E}_{\rm TI}(\cdot)e^{iH_{\rm B}t},\ \forall t\in\mathbb{R}. \tag{328}$$

Then, taking the average over t, we find that

$$\langle\psi|\mathcal{E}_{\rm TI}(\sigma)|\psi\rangle = d_{\rm B}\times\langle\gamma_{\rm BB'}|\mathcal{E}_{\rm TI}\otimes\mathcal{I}_{B'}(\Gamma_{\rm AB'})|\gamma_{\rm BB'}\rangle\ , \tag{329}$$

where

$$\Gamma_{\rm AB'} \equiv \lim_{T\to\infty}\frac{1}{T}\int_0^T dt\ \left([e^{iH_{\rm A}t}\otimes e^{-iH_{\rm B}t}][\sigma\otimes|\overline{\psi}\rangle\langle\overline{\psi}|][e^{-iH_{\rm A}t}\otimes e^{iH_{\rm B}t}]\right) \tag{330}$$

$$= \sum_E \Pi_E[\sigma_{\rm A}\otimes|\overline{\psi}\rangle\langle\overline{\psi}|_{\rm B}]\Pi_E\ , \tag{331}$$

where Π_E is the projector to the eigensubspace of $H_{\rm A}\otimes I_{\rm B} - I_{\rm A}\otimes H_{\rm B}$ with energy E.

Therefore,

$$\max_{\mathcal{E}_{\rm TI}}\langle\psi|\mathcal{E}_{\rm TI}(\sigma)|\psi\rangle = d_{\rm B}\times\max_{\mathcal{E}_{\rm TI}}\langle\gamma_{\rm BB'}|\mathcal{E}_{\rm TI}\otimes\mathcal{I}_{B'}(\Gamma_{\rm AB'})|\gamma_{\rm BB'}\rangle\ . \tag{332}$$

Next, we argue that in the right-hand side, instead of maximizing over the set of all TI operations from A to B, we can maximize over the larger set of *all* quantum operations from A to B (i.e. all CPTP maps) and still the equality remains valid. Let $\mathcal{E}$ be an arbitrary CPTP map from A to B. Using Supplementary Eq.(327) we have

$$\langle\gamma_{\rm BB'}|\mathcal{E}\otimes\mathcal{I}_{B'}(\Gamma_{\rm AB'})|\gamma_{\rm BB'}\rangle = \langle\gamma_{\rm BB'}|(e^{iH_{\rm B}t}\otimes e^{-iH_{\rm B}t})\mathcal{E}\otimes\mathcal{I}_{B'}(\Gamma_{\rm AB'})(e^{-iH_{\rm B}t}\otimes e^{iH_{\rm B}t})|\gamma_{\rm BB'}\rangle\ . \tag{333}$$

Next, we note that $\Gamma_{\rm AB'}$ has the symmetry,

$$\forall t\in\mathbb{R}:\quad [e^{-iH_{\rm A}t}\otimes e^{iH_{\rm B}t}]\Gamma_{\rm AB'}[e^{iH_{\rm A}t}\otimes e^{-iH_{\rm B}t}] = \Gamma_{\rm AB'}. \tag{334}$$

Combining this with Supplementary Eq.(333), we find

$$\langle\gamma_{\rm BB'}|\mathcal{E}\otimes\mathcal{I}_{B'}(\Gamma_{\rm AB'})|\gamma_{\rm BB'}\rangle = \langle\gamma_{\rm BB'}|(e^{iH_{\rm B}t}\otimes e^{-iH_{\rm B}t})\mathcal{E}\otimes\mathcal{I}_{B'}(\Gamma_{\rm AB'})(e^{-iH_{\rm B}t}\otimes e^{iH_{\rm B}t})|\gamma_{\rm BB'}\rangle \tag{335}$$

$$= \langle\gamma_{\rm BB'}|(e^{iH_{\rm B}t}\otimes e^{-iH_{\rm B}t})\mathcal{E}\otimes\mathcal{I}_{B'}\left([e^{-iH_{\rm A}t}\otimes e^{iH_{\rm B}t}]\Gamma_{\rm AB'}[e^{iH_{\rm A}t}\otimes e^{-iH_{\rm B}t}]\right)(e^{-iH_{\rm B}t}\otimes e^{iH_{\rm B}t})|\gamma_{\rm BB'}\rangle \tag{336}$$

$$= \langle\gamma_{\rm BB'}|(e^{iH_{\rm B}t}\otimes I_{B'})\mathcal{E}\otimes\mathcal{I}_{B'}\left([e^{-iH_{\rm A}t}\otimes I_{B'}]\Gamma_{\rm AB'}[e^{iH_{\rm A}t}\otimes I_{B'}]\right)(e^{-iH_{\rm B}t}\otimes I_{B'})|\gamma_{\rm BB'}\rangle, \tag{337}$$

where $I_{B'}$ is the identity operator on system B'. Taking the average over t, we find

$$\langle\gamma_{\rm BB'}|\mathcal{E}\otimes\mathcal{I}_{B'}(\Gamma_{\rm AB'})|\gamma_{\rm BB'}\rangle = \langle\gamma_{\rm BB'}|\tilde{\mathcal{E}}_{\rm TI}\otimes\mathcal{I}_{B'}(\Gamma_{\rm AB'})|\gamma_{\rm BB'}\rangle, \tag{338}$$

where we have defined

$$\tilde{\mathcal{E}}_{\rm TI}(X) \equiv \lim_{T\to\infty}\frac{1}{T}\int_0^T dt\ e^{iH_{\rm B}t}\mathcal{E}(e^{-iH_{\rm A}t}(X)e^{iH_{\rm A}t})e^{-iH_{\rm B}t}\ , \tag{339}$$

which is a TI operation.

This implies that in the right-hand side of Supplementary Eq.(332), maximization over TI quantum operations, can be replaced

by maximization over *all* quantum operations, i.e.

$$\max_{\mathcal{E}_{\text{TI}}}\langle\psi|\mathcal{E}_{\text{TI}}(\sigma)|\psi\rangle = d_{\text{B}} \times \max_{\mathcal{E}_{\text{TI}}}\langle\gamma_{\text{BB}'}|\mathcal{E}_{\text{TI}}\otimes\mathcal{I}_{B'}(\Gamma_{\text{AB}'})|\gamma_{\text{BB}'}\rangle \tag{340}$$

$$= d_{\text{B}} \times \max_{\mathcal{E}}\langle\gamma_{\text{BB}'}|\mathcal{E}\otimes\mathcal{I}_{B'}(\Gamma_{\text{AB}'})|\gamma_{\text{BB}'}\rangle. \tag{341}$$

Finally, using the result of [22] we note that

$$2^{-H_{\min}(\text{B}'|\text{A})_\Gamma} = d_{\text{B}} \times \max_{\mathcal{E}}\langle\gamma_{\text{BB}'}|\mathcal{E}\otimes\mathcal{I}_{B'}(\Gamma_{\text{AB}'})|\gamma_{\text{BB}'}\rangle\ , \tag{342}$$

where the maximization is over all CPTP maps from system A to system B'. Therefore, we conclude that

$$2^{-H_{\min}(\text{B}'|\text{A})_\Gamma} = \max_{\mathcal{E}_{\text{TI}}}\langle\psi|\mathcal{E}_{\text{TI}}(\sigma)|\psi\rangle\ . \tag{343}$$

Next, suppose in definition of state $\Gamma_{\text{AB}'}$, we replace $|\overline{\psi}\rangle\langle\overline{\psi}|$ with $|\psi\rangle\langle\psi|$, and define state

$$\Omega_{\text{AB}'} \equiv \lim_{T\to\infty}\frac{1}{T}\int_0^T dt\ \left([e^{iH_{\text{A}}t}\otimes e^{-iH_{\text{B}}t}][\sigma\otimes|\psi\rangle\langle\psi|][e^{-iH_{\text{A}}t}\otimes e^{iH_{\text{B}}t}]\right) \tag{344}$$

$$= \sum_E \Pi_E[\sigma_{\text{A}}\otimes|\psi\rangle\langle\psi|_{\text{B}}]\Pi_E\ , \tag{345}$$

where Π_E is the projector to the eigensubspace of $H_{\text{A}}\otimes I_{\text{B}} - I_{\text{A}}\otimes H_{\text{B}}$ with energy E.

Then, using Supplementary Eq.(343), we know that if we consider H_{min} for state $\Omega_{\text{AB}'}$ rather than state $\Gamma_{\text{AB}'}$, we find

$$2^{-H_{\min}(\text{B}'|\text{A})_\Omega} = \max_{\mathcal{E}_{\text{TI}}}\langle\overline{\psi}|\mathcal{E}_{\text{TI}}(\sigma)|\overline{\psi}\rangle\ . \tag{346}$$

Finally, we use the following fact, which is proven later: The maximum achievable fidelity with state $|\psi\rangle$ and state $|\overline{\psi}\rangle$ are equal, i.e.

$$\max_{\mathcal{E}_{\text{TI}}}\ \langle\psi|\mathcal{E}_{\text{TI}}(\sigma_{\text{A}})|\psi\rangle_{\text{B}} = \max_{\mathcal{E}_{\text{TI}}}\ \langle\overline{\psi}|\mathcal{E}_{\text{TI}}(\sigma_{\text{A}})|\overline{\psi}\rangle_{\text{B}}\ . \tag{347}$$

This combined with Supplementary Eq.(346) implies

$$\max_{\mathcal{E}_{\text{TI}}}\ \langle\psi|\mathcal{E}_{\text{TI}}(\sigma_{\text{A}})|\psi\rangle_{\text{B}} = \max_{\mathcal{E}_{\text{TI}}}\ \langle\overline{\psi}|\mathcal{E}_{\text{TI}}(\sigma_{\text{A}})|\overline{\psi}\rangle_{\text{B}} = 2^{-H_{\min}(\text{B}'|\text{A})_\Omega}\ , \tag{348}$$

which proves the theorem (Note that in the statement of theorem we have replaced label B' by B).

To complete the proof, we need to prove Supplementary Eq.(347), which is presented in the following: We use the fact that for any state $|\psi\rangle$ there exists a unitary V_ψ, which commutes with H_{B} and transforms $|\psi\rangle$ to $|\overline{\psi}\rangle = V_\psi|\psi\rangle$. In particular, we can choose

$$V_\psi = \sum_i \frac{\langle\psi|E_i\rangle}{\langle E_i|\psi\rangle}|E_i\rangle\langle E_i|, \tag{349}$$

where we assume $\frac{\langle\psi|E_i\rangle}{\langle E_i|\psi\rangle} = 1$ if $\langle\psi|E_i\rangle = 0$ (Note that, in general, this unitary depends on $|\psi\rangle$. A transformation which maps $|\psi\rangle$ to $|\overline{\psi}\rangle$ for all $|\psi\rangle$ should be anti-linear). Clearly, $[V_\psi, H_{\text{B}}] = 0$ and $V_\psi|\psi\rangle \equiv |\overline{\psi}\rangle$. Let $\mathcal{V}_\psi[\cdot] = V_\psi(\cdot)V_\psi^\dagger$ be the super-operator corresponding to the unitary V_ψ. Clearly $\mathcal{V}_\psi$ is a TI operation.

Next, we note that for any TI operation $\mathcal{E}_{\text{TI}}$,

$$\langle\psi|\mathcal{E}_{\text{TI}}(\sigma_{\text{A}})|\psi\rangle = \langle\overline{\psi}|\mathcal{V}_\psi\circ\mathcal{E}_{\text{TI}}(\sigma_{\text{A}})|\overline{\psi}\rangle \tag{350}$$

$$\langle\psi|\mathcal{V}_\psi^\dagger\circ\mathcal{E}_{\text{TI}}(\sigma_{\text{A}})|\psi\rangle = \langle\overline{\psi}|\mathcal{E}_{\text{TI}}(\sigma_{\text{A}})|\overline{\psi}\rangle\ . \tag{351}$$

Since both $\mathcal{V}_\psi$ and $\mathcal{V}_\psi^\dagger$ are TI operations, and TI operations are closed under composition, we conclude that both $\mathcal{V}_\psi^\dagger\circ\mathcal{E}_{\text{TI}}$ and $\mathcal{V}_\psi\circ\mathcal{E}_{\text{TI}}$ are also TI operation. Therefore, the above equations together imply

$$\max_{\mathcal{E}_{\text{TI}}}\ \langle\psi|\mathcal{E}_{\text{TI}}(\sigma_{\text{A}})|\psi\rangle_{\text{B}} = \max_{\mathcal{E}_{\text{TI}}}\ \langle\overline{\psi}|\mathcal{E}_{\text{TI}}(\sigma_{\text{A}})|\overline{\psi}\rangle_{\text{B}}\ . \tag{352}$$

This proves Supplementary Eq.(347) and completes the proof of the theorem.

□

Remark. This result can be easily extended to the case of symmetries described by a finite or compact Lie group G. Let $G \ni g \to U_{A/B}(g)$ be the unitary representations of symmetry G on the input system A and the output system B G. Then,

$$\max_{\mathcal{E}_{\text{cov}}} \langle\psi|\mathcal{E}_{\text{cov}}(\sigma_{\text{A}})|\psi\rangle_{\text{B}} = 2^{-H_{\min}(\text{B}|\text{A})_{\Gamma}} \ , \tag{353}$$

where the maximization is over the set of covariant operations, i.e. operations satisfying the covariance condition

$$\forall g \in G : \quad U_{\text{B}}(g)\mathcal{E}_{\text{cov}}(\cdot)\, U_{\text{B}}^{\dagger}(g) = \mathcal{E}_{\text{cov}}\left(U_{\text{A}}(g)(\cdot)U_{\text{A}}^{\dagger}(g)\right) \ , \tag{354}$$

and state

$$\Gamma_{\text{AB}} = \int dg\ [U_{\text{A}}(g) \otimes U_{\text{B}}(g)][\rho_{\text{A}} \otimes |\overline{\psi}\rangle\langle\overline{\psi}|_{\text{B}}][U_{\text{A}}^{\dagger}(g) \otimes U_{\text{B}}^{\dagger}(g)] \ . \tag{355}$$

Supplementary Note 10: Qubit example (Proof of Eq. 17 in the paper)

The smallest quantum clock is a qubit with two different energy levels. Without loss of generality we assume the Hamiltonian of this system is $H = \pi\sigma_z/\tau$. Suppose we want to prepare this clock in a state close to the pure state $|\Phi\rangle_{\text{c-bit}} = (|0\rangle + |1\rangle)/\sqrt{2}$, but we have access to the noisy version of this state, i.e. state

$$\rho = \lambda|\Phi\rangle\langle\Phi|_{\text{c-bit}} + (1-\lambda)I/2 \ , \tag{356}$$

with $0 < \lambda < 1$. The goal is to obtain a qubit state σ which has higher fidelity with $|\Phi\rangle_{\text{c-bit}}$, by combining n copies of this state via a TI operation. How close can we get to state $|\Phi\rangle_{\text{c-bit}}$? In other words, what is the maximum achievable fidelity,

$$\max_{\mathcal{E}_{\text{TI}}} \langle\Phi|\mathcal{E}_{\text{TI}}(\rho^{\otimes n})|\Phi\rangle_{\text{c-bit}} \ , \tag{357}$$

where the maximization is over all TI operations.

For any TI operation $\mathcal{E}_{\text{TI}}$, let $\sigma = \mathcal{E}_{\text{TI}}(\rho^{\otimes n})$ be the actual output state of the transformation. Then, using the monotonicity and the additivity of the purity of coherence, we find

$$P_H(\sigma) \leq P_{H_{\text{tot}}}(\rho^{\otimes n}) = n \times P_H(\rho) \ , \tag{358}$$

where $H_{\text{tot}} = \sum_i H^{(i)}$, and $H^{(i)} = I^{\otimes(i-1)} \otimes H \otimes I^{\otimes(n-i-1)}$.

As we saw in Supplementary Eq.(87), for a general qubit state ρ with the spectral decomposition $\rho = p|\psi\rangle\langle\psi| + (1-p)|\psi^\perp\rangle\langle\psi^\perp|$, the purity of coherence is given by

$$P_H(\rho) = \frac{(1-2p)^2}{p(1-p)} \times V_H(\psi) \ . \tag{359}$$

For state $\rho = \lambda|\Phi\rangle\langle\Phi|_{\text{c-bit}} + (1-\lambda)I/2$, we have $p = (1+\lambda)/2$, and $\psi = \Phi_{\text{c-bit}}$. Therefore,

$$P_H(\rho) = \frac{4\lambda^2}{1-\lambda^2} \times V_H(\Phi_{\text{c-bit}}) \ . \tag{360}$$

We conclude that for the output state σ, it holds that

$$P_H(\sigma) \leq \frac{4n\lambda^2}{1-\lambda^2} \times V_H(\Phi_{\text{c-bit}}) \ . \tag{361}$$

Next, we focus on the purity of coherence of the output state $\sigma = \mathcal{E}_{\text{TI}}(\rho^{\otimes n})$ and find a lower bound on $P_H(\sigma)$. Define state

$$\tilde{\sigma} = \frac{1}{2}(\sigma + X\sigma X) \ , \tag{362}$$

where $X = |0\rangle\langle 1| + |1\rangle\langle 0|$ is the Pauli-x operator. Using the fact that state $|\Phi_{\text{c-bit}}\rangle = (|0\rangle + |1\rangle)/\sqrt{2}$ is an eigenvector of X, it can be easily seen that the fidelity of $|\Phi_{\text{c-bit}}\rangle$ with states $\tilde{\sigma}$ and σ are equal, i.e.

$$\langle\Phi|\tilde{\sigma}|\Phi\rangle_{\text{c-bit}} = \langle\Phi|\sigma|\Phi\rangle_{\text{c-bit}} = \langle\Phi|\mathcal{E}_{\text{TI}}(\rho^{\otimes n})|\Phi\rangle_{\text{c-bit}} \ . \tag{363}$$

On the other hand, convexity of the purity of coherence implies

$$P_H(\tilde{\sigma}) = P_H(\frac{1}{2}[\sigma + X\sigma X]) \leq \frac{1}{2}P_H(\sigma) + \frac{1}{2}P_H(X\sigma X). \tag{364}$$

We can easily show that $P_H(X\sigma X) = P_H(\sigma)$. This follows, for instance, by noting that P_H is an even function of H, i.e. $P_H = P_{-H}$. Therefore $P_H(X\sigma X) = P_{-H}(X\sigma X) = P_{XHX}(X\sigma X)$, where we use the fact that the Pauli operator X, flips the sign of Hamiltonian $H = \pi\sigma_z/\tau$. Finally, we note that for any unitary U, $P_{UHU^\dagger}(U\sigma U^\dagger)$. Therefore, we conclude that $P_H(X\sigma X) = P_{-H}(X\sigma X) = P_{XHX}(X\sigma X) = P_H(\sigma)$, which implies

$$P_H(\tilde{\sigma}) = P_H(\frac{1}{2}[\sigma + X\sigma X]) \leq \frac{1}{2}P_H(\sigma) + \frac{1}{2}P_H(X\sigma X) = P_H(\sigma) \ . \tag{365}$$

Next, we note that $\tilde{\sigma}$ commutes with X, and therefore it can be written as

$$\tilde{\sigma} = \tilde{\lambda}|\Phi\rangle\langle\Phi|_{\text{c-bit}} + (1-\tilde{\lambda})I/2\ , \tag{366}$$

for some $0 \leq \tilde{\lambda} \leq 1$. This means that, given a fixed value of the purity of coherence, the state with this purity of coherence, which has the maximum fidelity with state $|\Phi\rangle_{\text{c-bit}}$ is in the form $\tilde{\lambda}|\Phi\rangle\langle\Phi|_{\text{c-bit}} + (1-\tilde{\lambda})I/2$.

Applying Supplementary Eq.(360) for state $\tilde{\sigma} = \tilde{\lambda}|\Phi\rangle\langle\Phi|_{\text{c-bit}} + (1-\tilde{\lambda})I/2$, we find

$$P_H(\tilde{\sigma}) = \frac{4\tilde{\lambda}^2}{1-\tilde{\lambda}^2} \times V_H(\Phi_{\text{c-bit}})\ . \tag{367}$$

Therefore, we conclude that

$$P_H(\sigma) \geq P_H(\tilde{\sigma}) = \frac{4\tilde{\lambda}^2}{1-\tilde{\lambda}^2} \times V_H(\Phi_{\text{c-bit}})\ . \tag{368}$$

Putting this into Supplementary Eq.(361) we find

$$\frac{\tilde{\lambda}^2}{1-\tilde{\lambda}^2} \leq n \times \frac{\lambda^2}{1-\lambda^2}\ . \tag{369}$$

which implies

$$\tilde{\lambda}^2 \leq \frac{n\lambda^2}{1+(n-1)\lambda^2} = \frac{1}{1+\frac{1}{n}(\frac{1}{\lambda^2}-1)}\ . \tag{370}$$

For a fixed $\lambda > 0$, in the large n limit this implies

$$\tilde{\lambda}^2 \leq \frac{1}{1+\frac{1}{n}(\frac{1}{\lambda^2}-1)} = 1 - \frac{1}{n}(\frac{1-\lambda^2}{\lambda^2}) + \mathcal{O}(\frac{1}{n^2})\ . \tag{371}$$

This means that, among all states whose purity of coherence are equal to the purity of coherence of the input, such that $P_H(\sigma) = n \times P_H(\rho)$, state $\sigma = \tilde{\lambda}|\Phi\rangle\langle\Phi|_{\text{c-bit}} + (1-\tilde{\lambda})I/2$, with

$$\tilde{\lambda} = \sqrt{1 - \frac{1-\lambda^2}{n\lambda^2} + \mathcal{O}(\frac{1}{n^2})} = 1 - \frac{1-\lambda^2}{2n\lambda^2} + \mathcal{O}(\frac{1}{n^2})\ , \tag{372}$$

has the minimum infidelity with state $|\Phi\rangle_{\text{c-bit}}$. This minimum infidelity is

$$1 - \langle\Phi|\tilde{\sigma}|\Phi\rangle_{\text{c-bit}} = \frac{1-\tilde{\lambda}}{2} = \frac{1-\lambda^2}{4n\lambda^2} + \mathcal{O}(\frac{1}{n^2})\ . \tag{373}$$

Therefore, for any TI process $\mathcal{E}_{\text{TI}}$,

$$1 - \langle\Phi|\mathcal{E}_{\text{TI}}(\rho^{\otimes n})|\Phi\rangle_{\text{c-bit}} \geq \frac{1-\lambda^2}{4n\lambda^2} + \mathcal{O}(\frac{1}{n^2})\ . \tag{374}$$

Remarkably, this bound is tight (up to a factor of 2). Using [23], we find that there exists a quantum operation $\mathcal{E}_{\text{Schur}}$ (related to the Schur transformation) which is covariant with respect to the full unitary group SU(2), for which the infidelity $1 - \langle\Phi|\mathcal{E}_{\text{Schur}}(\rho^{\otimes n})|\Phi\rangle_{\text{c-bit}}$ is equal to $2/(1+\lambda)$ times the right-hand side of this bound, i.e.

$$1 - \langle\Phi|\mathcal{E}_{\text{Schur}}(\rho^{\otimes n})|\Phi\rangle_{\text{c-bit}} = \frac{1-\lambda}{2n\lambda^2} + \mathcal{O}(\frac{1}{n^2})\ . \tag{375}$$

But, since this operation is covariant with respect to the full unitary group, it is also covariant with respect to time translations.

References:

[1] Marvian, I. Symmetry, Asymmetry and Quantum Information, PhD thesis. Ph.D. thesis, University of Waterloo, https://uwspace.uwaterloo.ca/handle/10012/7088, 2012.
[2] Aberg, J. Quantifying Superposition. *arXiv preprint quant-ph/0612146* **2006**,
[3] Gour, G.; Spekkens, R. W. The resource theory of quantum reference frames: manipulations and monotones. *New Journal of Physics* **2008**, *10*, 033023.
[4] Nielsen, M.; Chuang, I. *Quantum Computation and Quantum Information*; Cambridge Series on Information and the Natural Sciences; Cambridge University Press, 2000.
[5] Liu, Z.-W.; Hu, X.; Lloyd, S. Resource destroying maps. *Physical review letters* **2017**, *118*, 060502.
[6] Tomamichel, M. *Quantum Information Processing with Finite Resources: Mathematical Foundations*; Springer, 2015; Vol. 5.
[7] Petz, D. Quasi-entropies for finite quantum systems. *Reports on mathematical physics* **1986**, *23*, 57–65.
[8] Rudolph, T.; Spekkens, R. W.; Turner, P. S. Unambiguous discrimination of mixed states. *Physical Review A* **2003**, *68*, 010301.
[9] Berry, A. C. The accuracy of the Gaussian approximation to the sum of independent variates. *Transactions of the american mathematical society* **1941**, *49*, 122–136.
[10] Durrett, R. *Probability: theory and examples*; Cambridge university press, 2019; Vol. 49.
[11] Wilde, M. M. *Quantum information theory*; Cambridge University Press, 2013.
[12] Schuch, N.; Verstraete, F.; Cirac, J. I. Nonlocal resources in the presence of superselection rules. *Physical review letters* **2004**, *92*, 087904.
[13] Schuch, N.; Verstraete, F.; Cirac, J. I. Quantum entanglement theory in the presence of superselection rules. *Physical Review A* **2004**, *70*, 042310.
[14] Marvian, I. Coherence distillation machines are impossible in quantum thermodynamics. *arXiv preprint arXiv:1805.01989* **2018**,
[15] S.L. Braunstein and C.M. Caves, Statistical distance and the geometry of quantum states. **1994**, *72*, 3439.
[16] Barndorff-Nielsen, O.; Gill, R. Fisher information in quantum statistics. *Journal of Physics A: Mathematical and General* **2000**, *33*, 4481.
[17] Helstrom, C. W. Quantum detection and estimation theory. *Journal of Statistical Physics* **1969**, *1*, 231–252.
[18] A. S. Holevo, *Probabilistic and Statistical Aspects of Quantum Theory*; North-Holland: Amsterdam, 1982.
[19] Matsumoto, K. Reverse estimation theory, Complementality between RLD and SLD, and monotone distances. *arXiv preprint quant-ph/0511170* **2005**,
[20] Gour, G.; Jennings, D.; Buscemi, F.; Duan, R.; Marvian, I. Quantum majorization and a complete set of entropic conditions for quantum thermodynamics. *arXiv preprint arXiv:1708.04302* **2017**,
[21] Renner, R. Security of quantum key distribution. *International Journal of Quantum Information* **2008**, *6*, 1–127.
[22] Konig, R.; Renner, R.; Schaffner, C. The operational meaning of min-and max-entropy. *IEEE T. Inform. Theory* **2009**, *55*, 4337–4347.
[23] Cirac, J.; Ekert, A.; Macchiavello, C. Optimal purification of single qubits. *Physical review letters* **1999**, *82*, 4344.
[24] I thank an anonymous referee for pointing this out, which simplified the proof.
[25] Note that sometimes fidelity is defined as the square root of this formula.
